# Comprehensive Analysis of Universal Stress Protein Family Genes and Their Expression in *Fusarium oxysporum* Response of *Populus davidiana* × *P*. *alba* var. *pyramidalis* Louche Based on the Transcriptome

**DOI:** 10.3390/ijms24065405

**Published:** 2023-03-11

**Authors:** Jian Diao, Wei Gu, Zhehui Jiang, Jiaqi Wang, Hongfei Zou, Cheng Zong, Ling Ma

**Affiliations:** 1 College of Wildlife and Protected Area, Northeast Forestry University, Harbin 150040, China; 2 College of Forestry, Northeast Forestry University, Harbin 150040, China

**Keywords:** *Populus davidiana* × *P*. *alba* var. *pyramidalis* Louche, *Fusarium oxysporum*, USP gene family, plant–pathogen interaction, expression patterns of USPs

## Abstract

Universal stress proteins (USPs) are typical stress-inducible proteins that function directly in a variety of biotic or abiotic stresses and effectively protect plants from complex, adverse environments. However, the expression patterns of USP genes under pathogen stress and their molecular mechanisms in stress resistance have not been reported in detail. In this study, 46 USP genes were identified from *Populus trichocarpa* (PtrUSPs), and their biological characteristics were comprehensively analyzed based on phylogeny, physicochemical properties of proteins, and gene structures. The promoter regions of PtrUSPs contain a variety of cis-acting elements related to hormone and stress response. The results of a collinearity analysis showed that PtsrUSPs were highly conserved with homologous genes from four other representative species (*Arabidopsis thaliana*, *Eucalyptus grandis*, *Glycine max*, and *Solanum lycopersicum*). Furthermore, RNA-Seq analysis showed that the expression of 46 USPs from *P. davidiana* × *P. alba* var. *pyramidalis* Louche (PdpapUSPs) was significantly induced by *Fusarium oxysporum*. The co-expression network and gene ontology analysis of PtrUSPs showed that they participated in the response to stress and response to stimulus through precise coordination. The results of this paper systematically revealed the biological characteristics of PtrUSPs and the characteristics of their response to *F. oxysporum* stress, which will lay a theoretical foundation for improving genetic traits and the breeding of poplar disease-resistant varieties in subsequent studies.

## 1. Introduction

Universal stress proteins (USPs) were first discovered in 1990 [1,2]. They are typical stress-inducible proteins that are essential in various cellular responses under biotic or abiotic stress conditions such as nutrient deficiency [3], heat or cold stimulation [4], oxidative stress [5], and osmotic stress [4].

USPs are widely distributed in bacteria, fungi, plants, and animals, and are an important part of defense systems [6,7,8]. Studies showed that there are six subfamilies in the USP superfamily [9], namely UspA, UspC (yecG), UspD (yiiT), UspE (ydaA), UspF (ynaF), and UspG (ybdQ) [10,11]. Their protein sequences contain at least one USP signature domain (140–160 amino acids) and several motifs with other functions [12,13,14]. The sequence characteristics mean that USPs exhibit structural diversity and a variety of physiological and biochemical properties [1,7,15,16]. The physiological functions of USPs mainly include processes such as anti-oxidative stress, ion scavenging, and regulation of cell growth and development [17]. USPs also function in cell adhesion, agglutination, and cell motility [2,12]. Furthermore, USPs are significantly induced under adverse environmental conditions [1,18]. Some USPs also exhibit functions such as DNA binding, repair, and refolding activities; consequently, they can protect the nucleic acid structure in organisms from environmental stress [1,19]. USPs are widely distributed in plants such as *Brassica napus*, *Solanum lycopersicum*, *Oryza sativa*, *Populus trichocarpa*, and *Arabidopsis thaliana* [2,7,15]. Among them, *OsUSP1* in *O. sativa* was the first identified USP gene in plants, which is mainly involved in the activation of the ethylene signaling pathway during plant hypoxia [20]. In addition, USP genes in plants can also participate in regulatory processes such as the transportation of osmotic substances [21,22], stomatal pore size [4], antioxidant defense [22], and photosynthesis [4].

USPs in plants function under stress conditions, protecting plants from environmental stress, and providing a better likelihood of survival in complex environments [23]. *Phytophthora infestans* promoted the phosphorylation of protein kinases of *AtPHOS32* and *AtPHOS34* in *A. thaliana* and activated the transduction of plant defense signals to protect plants from pathogen attack [24,25]. *USPA* was resistant to fungi and parasites in *A. thaliana* and *Platycladus orientalis* (L.) Franco [26,27]. The USP gene *HRU1* in *A. thaliana* effectively regulated intracellular H_2_O_2_ content under hypoxic conditions, enabling plants to recover from hypoxia [28]. The overexpression of *AtUSP* (At3g53990) improved plant resistance to extreme temperatures or oxidative stress [23,29]. The expression level of *AtUSP* (At3g62550 and At3g53990) was significantly enhanced under drought conditions [30]. In addition, *AtUSP* (At3g53390) was significantly induced by salinity, osmotic stress, and trauma [31]. The overexpression of *GhUSP1* and *GhUSP2* in *Gossypium hirsutum* and *SpUSP* in *Solanum pennellii* conferred drought tolerance and effectively improved photosynthetic efficiency [4,11,32]. *SlRd2* in *S. lycopersicum* effectively improved tolerance to salt and osmotic stress in plants [33]. In addition, *SpUSP* was significantly induced by temperature extremes, mechanical damage, and phytohormones (abscisic acid, gibberellic acid, and ethylene) [4]. *OsUSP1* enhanced *O. sativa* resistance during flooding by regulating phytohormone signaling [20]. The ectopic expression of *SbUSP* in *Nicotiana tabacum* significantly enhanced tolerance to salt [34]. The modification of *GaUSP1* from *G. arboreum* improved drought tolerance in *G. hirsutum* [35]. *GhUSP2* regulated the growth state of plants under conditions of high temperature, osmotic pressure, and salt stress [36]. The overexpression of *MaUSP1* from *Morus alba* in *N. tabacum* enhanced its tolerance to drought, salinity, and oxidative stress [37]. In addition, USPs function directly in the growth and development of plants [2,23]. Some *USPA* genes are involved in the seed germination of *A. thaliana* [20,38,39]. USPs were shown to regulate fruit ripening by modulating ethylene-mediated signaling pathways [31]. The expression of *GUSP1* positively regulated the contents of proline, soluble sugar, and chlorophyll in plants [40].

USPs have important functions in plant physiology and metabolism, but the molecular mechanism of their roles in the resistance to stress is still unknown [2,41,42]. Therefore, analysis of characteristics and expression levels of USPs can provide theoretical support for gene function studies and the improvement of stress-tolerant crops. In this study, we systematically analyzed 46 USPs from *P. trichocarpa* (PtrUSPs), including cis-acting elements, chromosomal distribution, segmental duplication events, and collinearity analysis in related species. Furthermore, we identified the expression profile of USPs from *P. davidiana* × *P. alba* var. *pyramidalis* Louche (PdpapUSPs) infected by *F. oxysporum* based on RNA-Seq. Finally, we conducted a co-expression network of PdpapUSPs and revealed biological pathways related to various biological processes through gene ontology analysis. The research in this paper will enrich further studies on the functions and characteristics of poplar USPs, and lay a theoretical foundation for the next stage of disease resistance breeding in poplar.

## 2. Results

### 2.1. Phylogenetic Relationships and Physicochemical Properties of PtrUSPs

**Figure 1:**
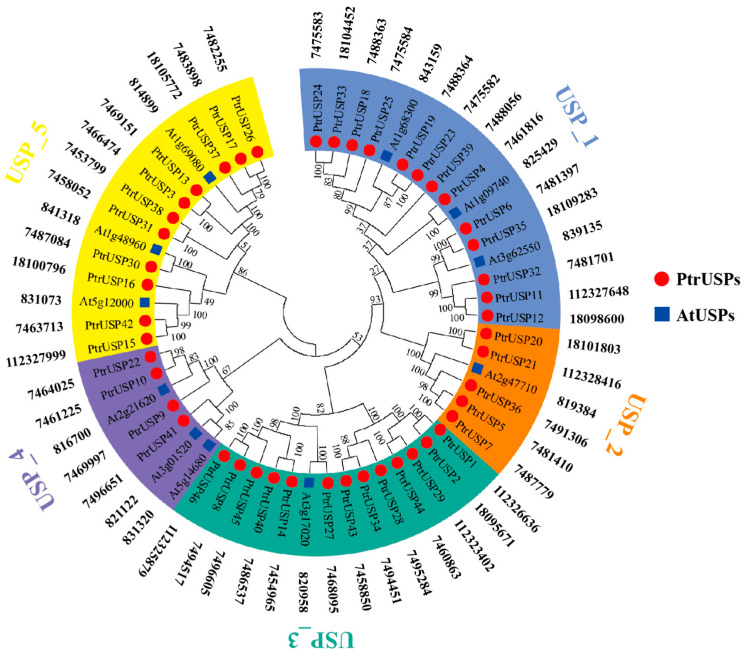
Phylogenetic analysis of USPs in *P. trichocarpa* and *A. thaliana*. Phylogenetic analysis of 57 USPs including *P. trichocarpa* and *A. thaliana* was performed by MEGA 5. ML method based on JTT + G + F model was used for phylogenetic analysis. Different groups were marked with different colors. USPs in this study were followed by gene IDs. Red dots represent PtrUSPs and blue boxes represent AtUSPs.

The 46 USPs identified in *P. trichocarpa* were named PtrUSPs in turn (serial numbers in this study were determined based on the position of each gene in the *P. trichocarpa* genome; Table S1). To compare evolutionary relationships among USP genes, a maximum likelihood (ML) phylogenetic tree was inferred using homologous sequences of USPs in *P. trichocarpa* and *A. thaliana* (Figure 1). The results showed that PtrUSPs were divided into five groups with different sizes according to the evolutionary relationships and functions of conserved domains in sequences. Different groups were marked with different colors. USP_1 and USP_3 are the largest, each containing 13 PtrUSPs. USP_4 is the smallest, containing only four PtrUSPs. USP_2 contains 5 PtrUSPs, and USP_5 contains 11 PtrUSPs. In addition, homologous genes of USPs in *A. thaliana* were randomly distributed in each group.

The physicochemical properties of PtrUSPs vary greatly, but the properties of members in the same group were basically the same (Table S1). The average length of PtrUSPs was 234 amino acids (range = 157–774 aa) and the average molecular weight was 25.80 kDa (range = 17.36–85.94 kDa). The total number of atoms in PtrUSPs ranged from 2483 to 12,069 and the range of theoretical isoelectric point (pI) was 4.84–10.76. The aliphatic index ranged from 75.19 to 106.60. The hydrophilicity values of PtrUSPs ranged from −0.676 to 0.225, and PtrUSPs are likely to have great differences in hydrophilicity and hydrophobicity. The instability indexes of PtrUSPs ranged from 22.86 to 56.91 (“>40” means proteins are unstable, and “<40” means proteins are stable).

### 2.2. Gene Structure Analysis of PtrUSPs

**Figure 2:**
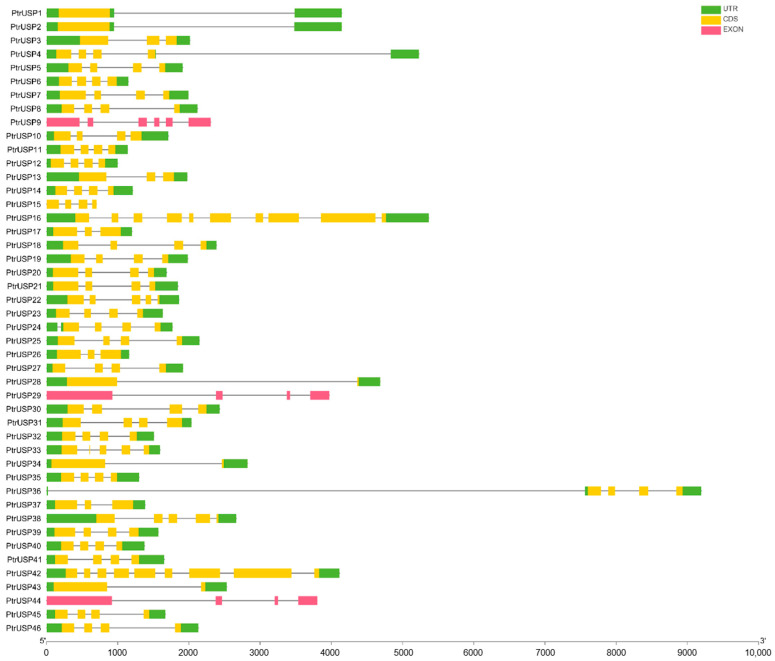
Gene structures of members in PtrUSPs. Green boxes represent untranslated regions and yellow boxes represent coding regions. Pink boxes represent exons and black lines represent introns.

PtrUSP sequences in the same group were highly similar in the composition of exons and introns. In addition, members that were more closely related shared more similar exon lengths and gene structures (Figure 2). Except for *PtrUSP33*, which contains 5 exons, the other 12 members in the group USP_1 all contain 4 exons. All members in the group USP_2 contain four exons. Eight members in the group USP_3 contain four exons, and the remaining five members contain one or two exons. The gene structures of members in the group USP_4 and the group USP_5 were different, but the structures of members with a close evolutionary relationship were similar.

**Figure 3:**
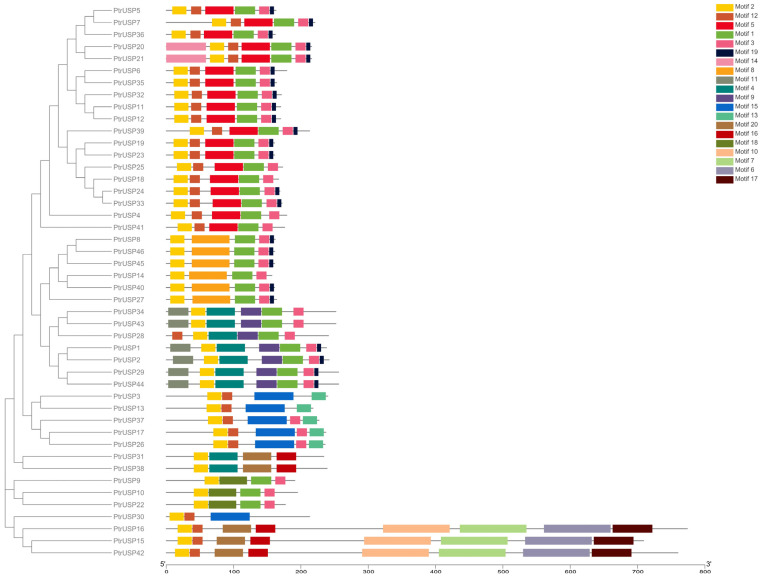
Protein motifs of PtrUSPs. Boxes of different colors represent different motifs.

Twenty conserved domains in PtrUSPs were identified by MEME, and annotation information of motifs were predicted by Pfam and InterProScan (Table S2). The results showed that motifs 1, 2, 3, 4, and 15 were annotated as USP family proteins. Motif 6 was annotated as a protein kinase domain. Motif 7 was annotated as protein tyrosine and serine/threonine kinase. Motif 10 was annotated as PAXX, PAralog of XRCC4 and XLF, also called C9orf142. Motif 16 was annotated as sporulation lipoprotein YhcN/YlaJ (Spore_YhcN_YlaJ). Motif 17 was annotated as a protein of unknown function (DUF3012). No annotation information was obtained for motifs 5, 8, 9, 11, 12, 13, 14, 18, 19, or 20. An overall analysis of conserved domains in PtrUSPs revealed that motif 2 is present in all PtrUSPs (Figure 3). Motifs 6, 7, 10, 15, 16, and 17 are only present in members of USP_5. In addition, PtrUSPs with a close evolutionary relationship have highly identical or similar features in the composition of conserved domains. These results showed that different motifs in PtrUSPs may be related to various, specific biological functions of PtrUSPs.

### 2.3. Secondary and Tertiary Structure Analysis of PtrUSPs

In secondary structures of PtrUSPs (Table S3), an average of 82 amino acids (AAs) constituted α helices, accounting for 37.76% of the total secondary structures. On average, 40 AAs (18.89%) constituted extended strands and 12 (5.57%) constituted beta turns. In addition, 88 AAs (37.79%) constituted random coils.

The tertiary structures of PtrUSPs were mainly templates 1mjh.1.A, 2gm3.1.A, and 3hgm.1.A (Table S4). The main functions of templates are USP TeaD and ehCrystal structure of a USP family protein from *A. thaliana* At3g01520 with bound AMP. The average sequence identity of PtrUSPs to their corresponding templates was 24.55% (10.64–72.99%).

### 2.4. Topological Heterogeneity Model and Subcellular Localization Analysis of PtrUSPs

Results of topological heterogeneity models showed that no PtrUSPs contained transmembrane helical regions (Table S5). A total of 27 PtrUSPs contained N-glycosylation sites. Furthermore, only PtrUSP20 and PtrUSP21 had signal peptides.

The results of subcellular localizations indicated that most PtrUSPs were located in the cytoplasm (Table S6). PtrUSP1, 2, 3, 16, 33, 38, and 42 were located in the nucleus and PtrUSP20, 21, 24, 25, 28, 41, and 44 were located in chloroplasts. PtrUSP18 was located in the peroxisome and PtrUSP37 was located in the mitochondria.

### 2.5. Cis-Acting Elements Analysis in Promoters of PtrUSPs

**Figure 4:**
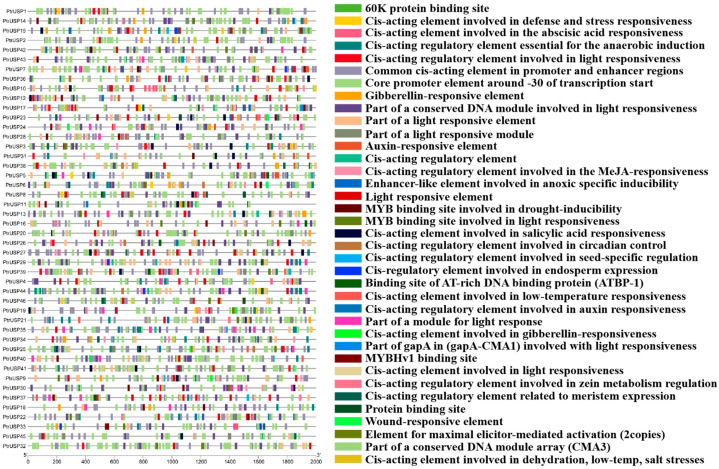
Analysis of cis-acting elements in promoters of PtrUSPs. Boxes of different colors represent different cis-acting elements.

The results showed that cis-acting elements in promoters are involved in plant responses to various hormones such as abscisic acid, gibberellin, auxin, MeJA, and salicylic acid (Figure 4). Many elements are also involved in plant defense and stress responses (such as hypoxia, drought, low temperature, injury, and salt stress), light response, circadian rhythm control, seed-specific regulation, endosperm expression, meristem expression, and other processes.

### 2.6. Chromosomal Localization and Collinearity Analysis of PtrUSPs

**Figure 5:**
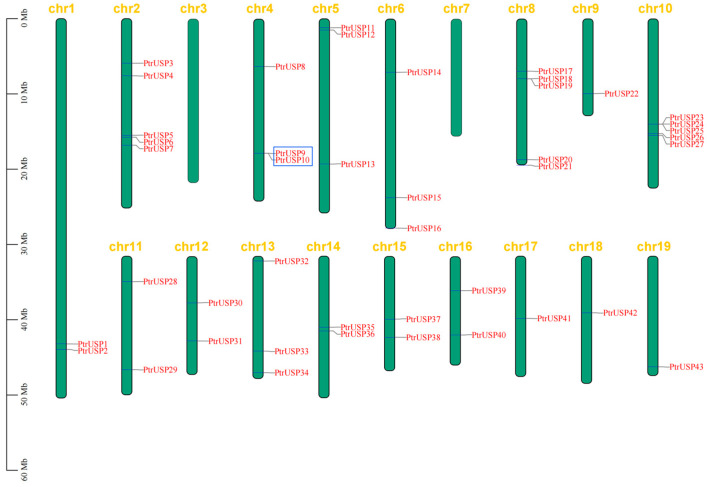
Chromosomal distribution of PtrUSPs. Green boxes represent chromosomes and blue boxes represent gene pairs with tandem repeats. Chr1–19 represent chromosome numbers 1–19.

The chromosomal distribution of PtrUSPs is random and variable (Figure 5) and they were distributed on 17 of the 19 poplar chromosomes. The number of genes distributed on each chromosome was independent of chromosome size. The number of PtrUSPs distributed on chromosomes 2, 8, and 10 was the largest, with 5 genes each. Only one *PtrUSP* was distributed on each of the chromosomes 9, 17, 18, and 19. None were distributed on chromosomes 3 and 7.

**Figure 6:**
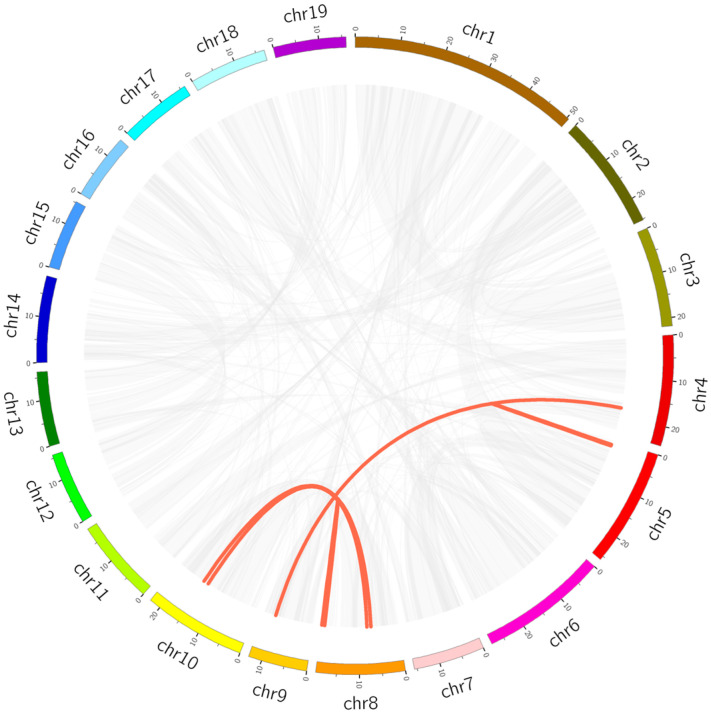
Segmental duplication events of PtrUSPs. Boxes with different colors represent different chromosomes and Chr1–19 represent chromosome numbers 1–19. Red lines represent collinear pairs of PtrUSPs and grey lines represent all collinear pairs in *P. trichocarpa* genome.

The duplication events of PtrUSPs in the poplar genome were analyzed by MCScanX. *PtrUSP9* and *PtrUSP10* (distributed on chromosome 4) were identified as tandem duplication events (Figure 5). In addition, 11 genes exhibited 6 pairs of segmental duplication events (Figure 6, Table S7). These genes were shown to be randomly distributed on 5 of the 19 chromosomes. The mean value about ratios of non-synonymous substitution to synonymous substitution (Ka/Ks) for segmental duplication events in PtrUSPs was 0.23 (range from 0.07 to 0.46), which suggested that PtrUSPs were subjected to purifying selection during evolution.

**Figure 7:**
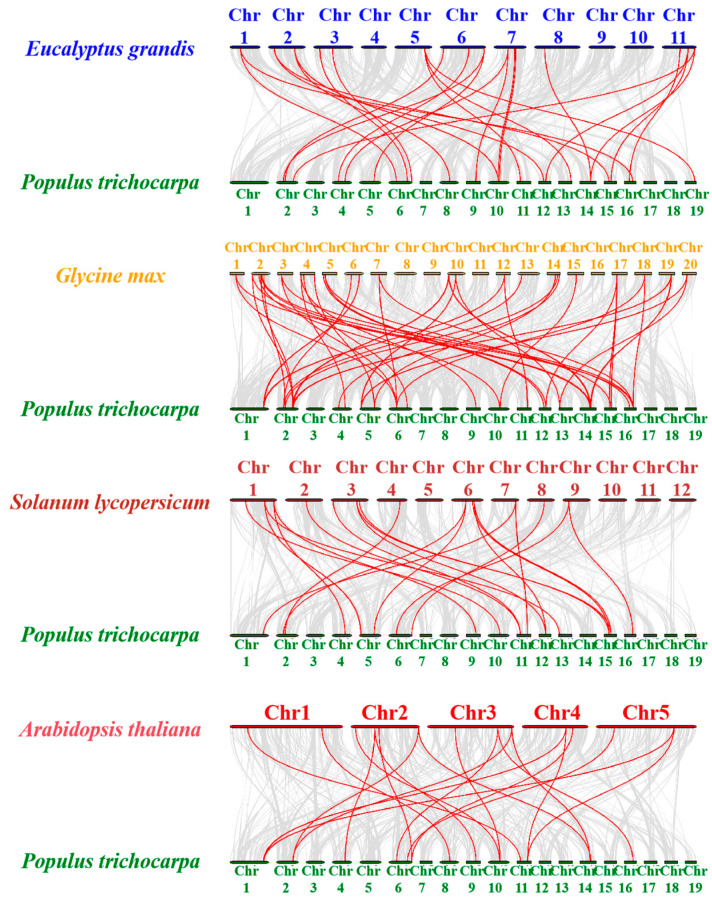
Collinearity relationship map between PtrUSPs and homologous genes in 4 species (*G. max*, *E. grandis*, *S. lycopersicum*, and *A. thaliana*). Red lines represent the collinearity between PtrUSPs and homologous genes in other species. Grey lines represent orthologous collinearity between *P. trichocarpa* genome and genomes of other species. Chr1–19 represent chromosome numbers 1–19.

To reveal the similarity between PtrUSPs and homologous genes in other species, we constructed separate collinearity relationship maps between *P. trichocarpa* and four representative dicotyledonous species (*G. max*, *E. grandis*, *S. lycopersicum*, and *A. thaliana*) (Figure 7). The results showed that there were 50 repetitive events in *G. max*, 28 in *E. grandis*, 22 in *S. lycopersicum*, and 21 in *A. thaliana* (Table S8).

### 2.7. Expression Patterns of PdpapUSPs in Response to F. oxysporum

**Figure 8:**
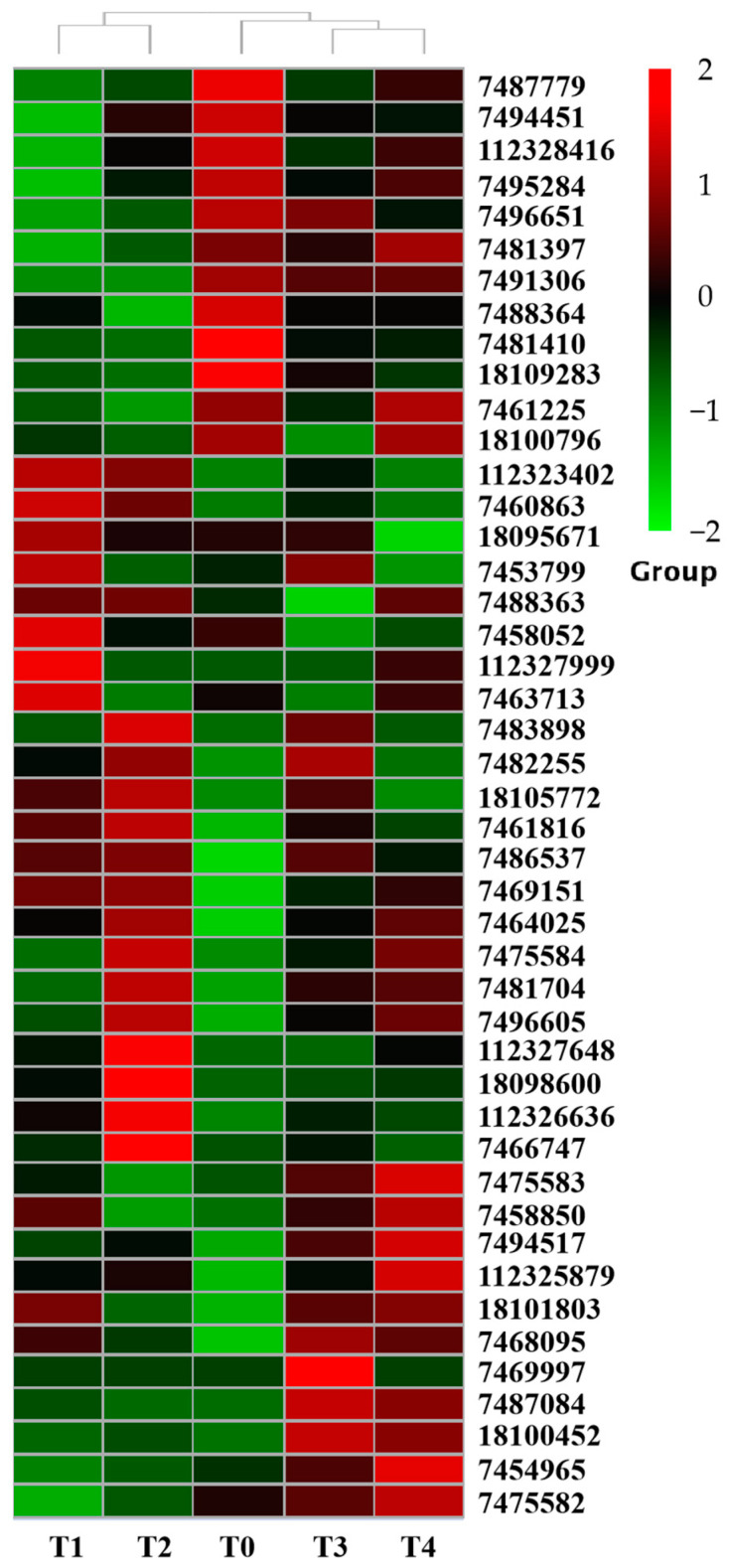
Cluster analysis of expression levels in PdpapUSPs treated with *F. oxysporum* at different times. Cluster analysis was based on log_2_FPKM at different times. Red boxes represent highly expressed genes and green boxes represented low expressed genes. Left side represents gene clusters. T0, T1, T2, T3, and T4 indicated 0, 6, 12, 24, and 48 h after treatment of Pdpap by *F. oxysporum*, respectively.

The expression patterns of PdPapUSPs infected by *F. oxysporum* at different times (6 h, 12 h, 24 h, and 48 h) were analyzed by RNA-Seq (Table S9). The expression levels of PdPapUSPs were different under different treatments and they were significantly induced by *F. oxysporum* (Figure 8). The fragments per kilobase of exon per million mapped fragments (FPKM) values of PdPapUSPs for each treatment are shown in Table S9.

**Figure 9:**
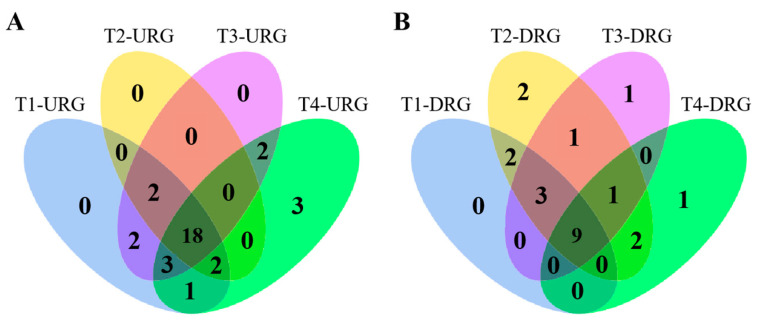
Venn diagram of DEG expression levels under different *F. oxysporum* treatments. (**A**) Venn diagram of up-regulated DEGs (URGs) under different *F. oxysporum* treatments. (**B**) Venn diagram of down-regulated DEGs (DRGs) under different *F. oxysporum* treatments.

The numbers of up- and down-regulated differentially expressed genes (DEGs) in four treatment groups (6 h, 12 h, 24 h, and 48 h) were visualized by separate Venn diagrams (Figure 9). The results showed that 18 genes were up-regulated in Pdpap after treatment with *F. oxysporum* among the four treatments and nine genes were down-regulated in Pdpap. The differential expression fold changes of PdpapUSPs in each treatment are shown in Table S10.

**Figure 10:**
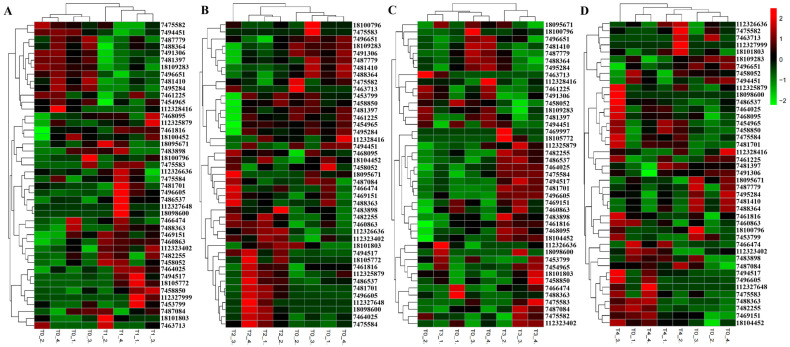
Cluster analysis of DEG expression levels under different *F. oxysporum* treatments. (**A**) Cluster analysis of DEG expression levels after 6 h of *F. oxysporum* treatment. (**B**) Cluster analysis of DEG expression levels after 12 h of *F. oxysporum* treatment. (**C**) Cluster analysis of DEG expression levels after 24 h of *F. oxysporum* treatment. (**D**) Cluster analysis of DEG expression levels after 48 h of *F. oxysporum* treatment. Cluster analysis was based on log_2_FPKM. Red boxes represent highly expressed genes and green boxes represent low expressed genes. Left side represents gene clusters. T0, T1, T2, T3, and T4 represent Pdpap treated by *F. oxysporum* for 0, 6, 12, 24, and 48 h, respectively.

The expression levels of DEGs in each treatment were analyzed by cluster analysis (Figure 10). Differential expression fold changes of genes in each treatment are shown in Table S10. After 6 h of *F. oxysporum* treatment, 44 DEGs were identified, including 30 up-regulated genes and 14 down-regulated genes (Figure 10A). Among them, *PdPapUSP8* had the highest up-regulation (4.49×), and *PdPapUSP21* had the highest down-regulation (−2.38×). After 12 h of *F. oxysporum* treatment, 42 DEGs were identified, including 22 up-regulated genes and 20 down-regulated genes (Figure 10B). Among them, the highest up-regulation was identified in *PdPapUSP12* (5.76×), while *PdPapUSP5* had the highest down-regulation (−2.19×). After 24 h of *F. oxysporum* treatment, 42 DEGs were identified, including 27 up-regulated genes and 15 down-regulated genes (Figure 10C). Among them, the up-regulation of *PdPapUSP8* was the highest (5.88×), and the highest down-regulation was seen in *PdPapUSP18* (−2.15×). After 48 h of *F. oxysporum* treatment, 42 DEGs were identified, including 29 up-regulated genes and 13 down-regulated genes (Figure 10D). Among them, the highest up-regulation was observed in *PdPapUSP8* (6.91×), and the highest down-regulation was seen in *PdPapUSP5* (−1.50×).

### 2.8. Validation of PtrUSP Expression Levels

**Figure 11:**
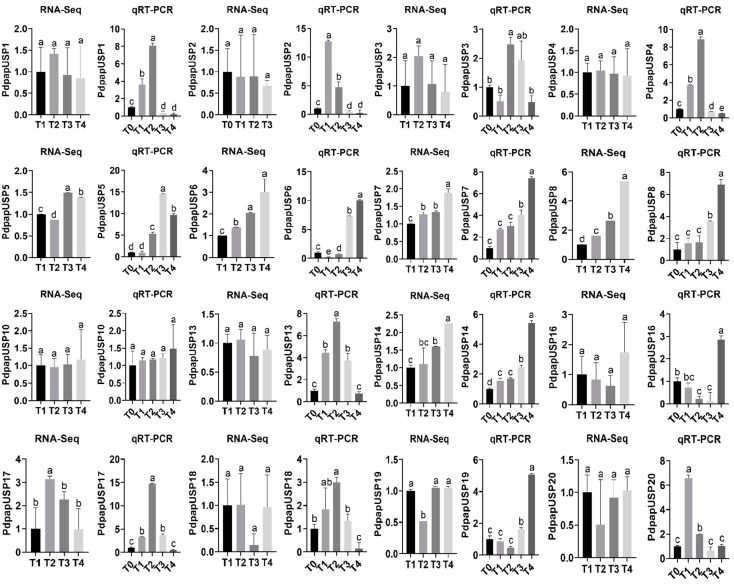

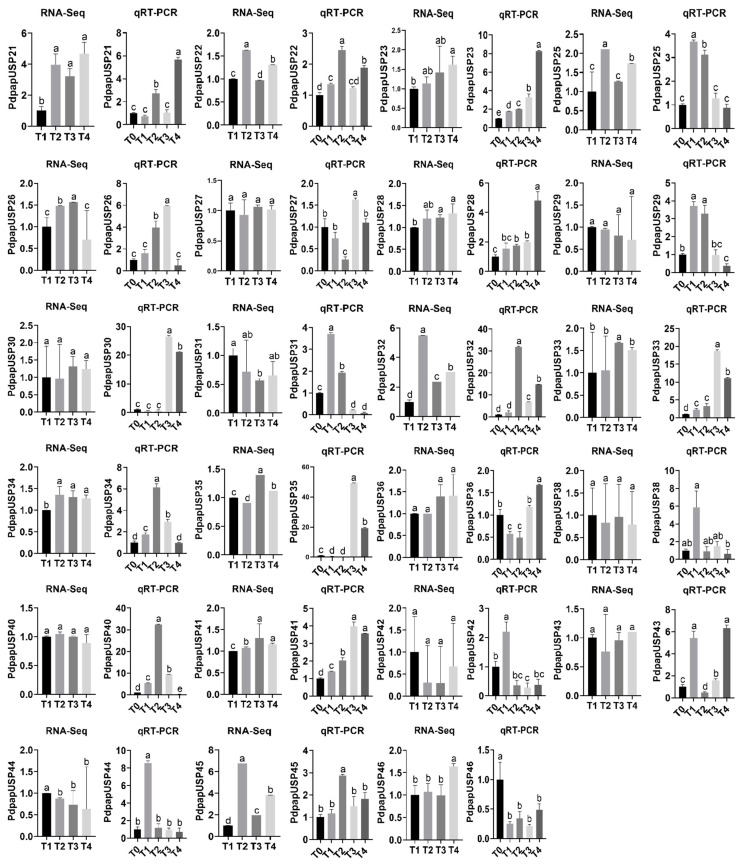
Comparison of PdpapUSPs expression levels by RNA-Seq and qRT-PCR. Expression levels in RNA-Seq were quantified by fragments per kilo-bases per million mapped reads (FPKM). T0, T1, T2, T3, and T4 represent PdPap infected by *F. oxysporum* for 0, 6, 12, 24, and 48 h, respectively. Error bars represent standard errors of three independent replicates and different lowercase letters represent the degree of significant difference (*p* < 0.05).

To verify the dependence of RNA-Seq in Pdpap treated by *F. oxysporum*, we analyzed expression levels of PdpapUSPs under each treatment by quantitative real-time PCR (qRT-PCR). The results showed that under different treatments, the expression trends of genes in RNA-Seq and qRT-PCR were basically similar, except for *PdPapUSP2*, *PdPapUSP5*, *PdPapUSP10*, *PdPapUSP13*, *PdPapUSP18*, and *PdPapUSP31*. The differences in expression trends of some genes may be due to experimental errors in RNA-Seq or qRT-PCR (Figure 11). A comparison of RNA-Seq and expression levels of PdpapUSPs under different treatments showed that *PdPapUSP8*, *PdPapUSP32*, and *PdPapUSP45* had higher expression levels in Pdpap.

### 2.9. Gene Co-Expression Analysis

**Figure 12:**
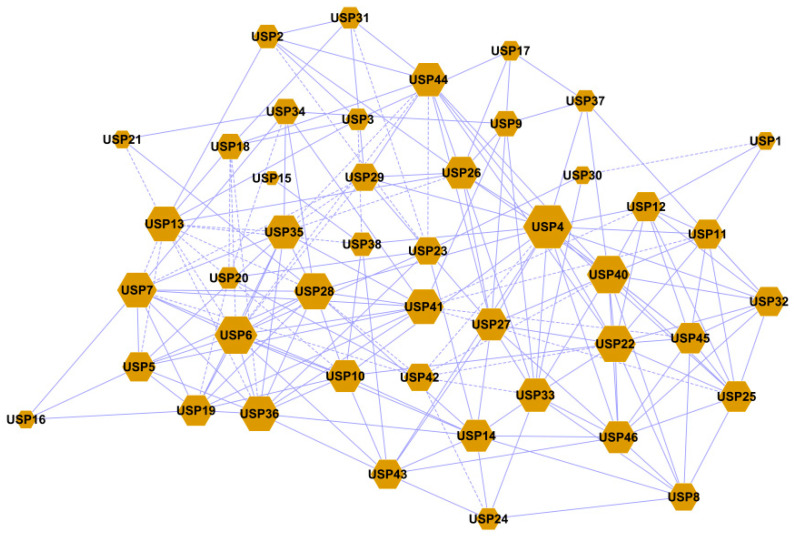
Co-expression network analysis of PdpapUSPs. Yellow dots represent PdpapUSPs and blue lines represent co-expression relationships among genes.

A co-expression network analysis was performed based on RNA-Seq of 46 PdPapUSPs (Figure 12). The results showed that PdPapUSPs were interconnected and cooperated to form a complex regulatory network. Genes such as *PdpapUSP4*, *PdpapUSP6*, *PdpapUSP22*, *PdpapUSP28*, *PdpapUSP36*, and *PdpapUSP40* occupy important positions in the network.

### 2.10. Gene Ontology (GO) Analysis

**Figure 13:**
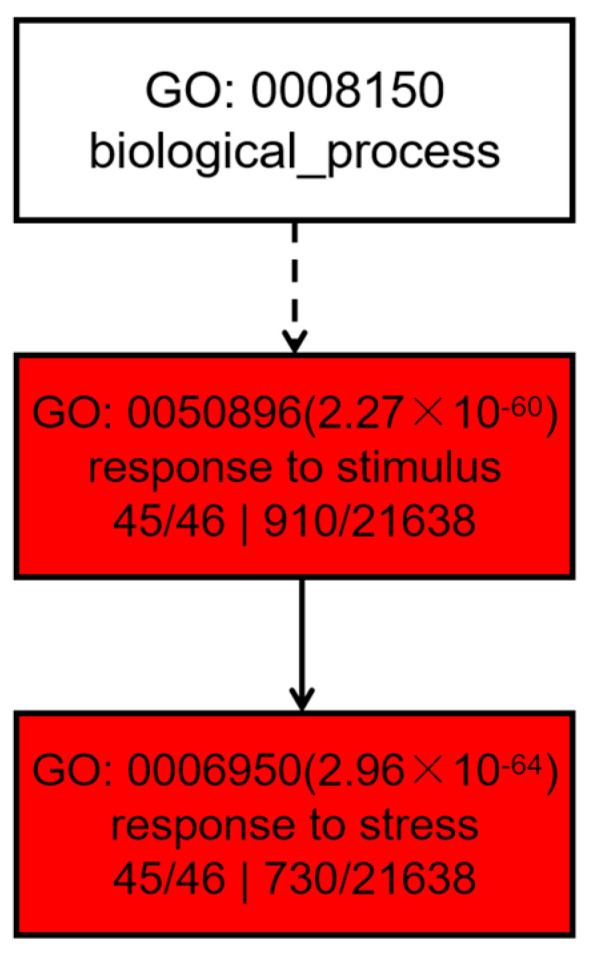
GO analysis of PtrUSPs based on biological process.

A gene enrichment analysis of PtrUSPs was performed by agriGO (Figure 13). The enrichment results showed that PtrUSPs were mainly involved in two types of biological processes: response to stress and response to stimulus (Table S11). The prediction results confirmed that almost all PtrUSPs were related to the response process to stress.

## 3. Discussion

USPs are widely distributed in a variety of organisms and are an important part of defensive systems [6,7,8]. In plants, USPs are directly involved in the process of resisting environmental disturbances and in numerous physiological and metabolic activities [2,12]. Research on the functions and characteristics of USPs could lay a theoretical foundation for genetic improvement and resistance breeding. Among the 13 PtrUSPs that are homologous to *A. thaliana*, 12 PtrUSPs and their homologous genes in *A. thaliana* are very close in evolutionary distance. Only *PtrUSP40* and its homologous gene At5g14680 are far apart in evolutionary distance and are in different groups. That may be because *PtrUSP40* and At5g14680 are paralogous genes. Studies have shown that paralogous gene differentiation caused by gene duplication can promote genes producing new properties or functions. They also differ in sequences and functions over time. That may be one of the main reasons for functional differences among homologous genes in different species [43,44].

PtrUSPs differ greatly in the composition of exons and introns. Genes in the same group have minimal differences in gene structures, and members with similar evolutionary relationships share similar gene structures. Gene structures of members in USP_1 and USP_2 are highly similar. Although the gene structures of members in other groups are quite different, genes with similar evolutionary relationships still maintain high unity. The number of introns is positively correlated with the time it takes for transcription to translation of a gene. The lower the number of introns, the faster the gene can be expressed during environmental changes, so that it can function more efficiently [45]. The results showed that only USP_3 has several genes with a small number of introns. Therefore, we speculated that genes in USP_3 may have more important functions in responding to environmental stress. Focusing on genes in group USP_3 could lead to more targeted research on resistant varieties.

Similar to the characteristics of gene structures, proteins with close evolutionary relationships are more similar in the composition of conserved domains. All PtrUSPs contain USP family protein domains. In addition to the family-characterized domains, most proteins also contain a protein kinase domain, protein tyrosine and serine/threonine kinase, C9orf142, and sporulation lipoprotein YhcN/YlaJ (Spore_YhcN_YlaJ) domains. Additionally, all proteins contained several domains without annotation information. The structural diversity of protein domains may be one of the main reasons for the diverse physiological and metabolic functions of PtrUSPs [46].

No PtrUSPs contain transmembrane helical regions. Except for PtrUSP20 and PtrUSP21 with signal peptides that can function outside the membrane, the remaining 44 proteins function in the membrane. That is because, under the action of signal peptides, PtrUSP20 and PtrUSP21 will be directed to organelles containing membrane structures that allow them to function. In total, 27 proteins in PtrUSPs contain N-glycosylation sites. N-glycosylation sites can be essential in protein folding and material transport [47]. Therefore, proteins containing N-glycosylation sites may transport foreign toxic substances and defense substances produced by plants out of host cells, thereby enabling plants to develop effective resistance to environmental stress [48,49,50].

Cis-acting elements in promoter regions can have an important role in regulating gene expression [51]. By analyzing cis-acting elements, genes with specific functions related to plant stress resistance, growth, and development can be effectively screened. The analysis of promoter regions showed that there were cis-acting elements related to the hormone response and stress response of PtrUSPs. In addition, many elements are involved in various aspects of plant growth and development. Therefore, PtrUSPs are crucial in regulating the defense of poplar against different environmental stresses. In addition, PtrUSPs can effectively regulate the growth and development of poplar, thereby ensuring normal growth and state recovery of plants after environmental stress.

Poplar has undergone at least three rounds of whole-genome duplication in addition to segmental duplication, tandem duplication, and transposition events [52,53]. Under the action of segmental duplication, tandem duplication, and transition events, genes can differentiate and generate multiple subfamilies [54,55]. The emergence of new genes after gene differentiation can effectively increase the diversity of gene functions in a gene family, thereby significantly improving the adaptability of plants to environmental stress [56]. In this study, we analyzed the duplication events of PtrUSPs in the *P. trichocarpa* genome. Segmental duplication events (six pairs) occurred more frequently than tandem duplication events (one pair). Compared with tandem duplication events, segmental duplication events may be the main reason for the diversity of PtrUSPs. Segmental duplication events might have a more important role in the expansion of diversity and functions of PtrUSPs. Ka/Ks is the main indicator for judging whether there was selective pressure acting on protein-coding genes during evolution. Ka/Ks < 1 means there was a purifying selection. Ka/Ks = 1 means there was a neutral selection. Ka/Ks > 1 means there was a positive selection. After analyzing Ka/Ks of segmental duplication events in PtrUSPs, some PtrUSPs have clearly undergone a purifying selection process during evolution.

Species with closer evolutionary relationships have more collinear gene pairs [57]. In order to reveal the similarity and evolutionary relationships of USPs among different species, collinear relationships among PtrUSPs and homologous genes in four other representative dicotyledonous plants were analyzed. The results showed that PtrUSPs had the highest homology with USPs in *G. max*, and relatively lower homology with USPs in *A. thaliana*. Therefore, based on functional studies of USPs in *G. max*, *E. grandis*, *S. lycopersicum*, and *A. thaliana*, theoretical support can be provided for subsequent screening and functional studies of key genes in PtrUSPs.

RNA-Seq showed that the expression of PdPapUSPs was significantly induced under *F. oxysporum* infection. A total of 18 genes in PdpapUSPs showed up-regulation, and 9 genes showed down-regulation. By analyzing RNA-Seq values and expression levels of genes under different treatments, it was found that *PdpapUSP8*, *PdpapUSP32*, and *PdpapUSP45* showed significant responses under *F. oxysporum* infection. They showed a positive response to the induction of *F. oxysporum* infection. According to follow-up experiments and expression patterns of USPs in other plants and microbial species [27,58,59], USPs can be used as key resistance genes for functional research to effectively promote the improvement of genetic traits and breeding of resistant varieties. In addition, results of gene enrichment analysis showed that PtrUSPs mainly functioned in two biological processes: response to stress and response to stimulus. Combined with previous results, USPs can be speculated to function directly in the response regulation of Pdpap under *F. oxysporum* infection.

Numerous members of a gene family can function through complex inter-regulation processes and act in the same signaling pathways or physiological processes [60]. Gene co-expression analysis can be used to intuitively understand genes with similar expression patterns in PdpapUSPs, and conduct a comprehensive analysis of complex regulatory networks among genes, to further explore functional correlations among PdpapUSPs. The results of co-expression networks in this paper confirmed close relationships among PdpapUSPs. Based on previous results, the expression patterns of genes in the co-expression network are quite different. Thus, regulatory profiles of USPs in response to *F. oxysporum* are extremely complex and variable.

Previous studies have shown that USPs are mainly involved in the regulatory process related to stress response [22]. Based on that, we speculated that they might function in enhancing the resistance response of poplar to *F. oxysporum* infection. The results of this study systematically analyzed the biological characteristics and expression patterns of USPs. Subsequent research will focus on the functional study of genes related to pathogen resistance, and further realize the selection and breeding of poplar disease-resistant varieties.

## 4. Materials and Methods

### 4.1. Screening and Identification of USPs in P. trichocarpa

Genomic data of *P. trichocarpa* was downloaded from Phytozome v12.1 (https://phytozome.jgi.doe.gov/pz/portal.html, accessed on 4 May 2021) [61] and then scanned by HMMER3 to find potential PtrUSPs [62]. Potential PtrUSPs were validated using the SMART database (http://smart.embl-heidelberg.de/, accessed on 12 May 2021) [63] and the Pfam database (http://Pfam.xfam.org, accessed on 12 May 2021) [64] to remove proteins without USP domains.

### 4.2. Evolutionary Relationship and Physicochemical Properties of USPs

USP sequences in *P. trichocarpa* and *A. thaliana* were separately downloaded from Phytozome. The phylogenetic tree of USPs was constructed in MEGA v5.1 [65] using ML, and the model was JTT (protein mutation data matrix) + G (site-specific variations in mutation rate) + F (mutation frequency data) [66].

Physicochemical properties of PtrUSPs were predicted by ProtParam (https://web.expasy.org/protparam/, accessed on 21 May 2021) [67], including number of amino acids, molecular weight, chemical formula, and instability index.

### 4.3. Gene Structure and Protein Motif Analysis of PtrUSPs

DNA and protein-coding sequences (CDSs) of PtrUSPs were downloaded from Phytozome. DNA and CDSs were aligned to obtain gene structures of PtrUSPs, and the gene structures were visualized in TBtools [68].

Conserved motifs in PtrUSPs were identified by MEME (http://meme-suite.org/, accessed on 28 May 2021) [69] and annotation information was obtained for identified motifs from InterProScan (https://www.ebi.ac.uk/interpro/search/sequence/, accessed on 4 June 2021) [70].

### 4.4. Secondary and Tertiary Structure Analysis of PtrUSPs

Secondary structures of PtrUSPs were predicted by SOPMA (https://npsa-prabi.ibcp.fr/cgi-bin/npsa_automat.pl?page=npsa_sopma.html, accessed on 13 June 2021) [71].

Tertiary structure models of PtrUSPs were constructed by SWISS-MODEL (https://swissmodel.expasy.org/, accessed on 17 June 2021) [72].

### 4.5. Topological Heterogeneity Model and Subcellular Localization Prediction of PtrUSPs

Topological heterogeneity models of PtrUSPs were predicted by Protter (http://wlab.ethz.ch/protter/start/, accessed on 27 June 2021) [73].

Subcellular localization of PtrUSPs was predicted by WoLF PSORT (https://wolfpsort.hgc.jp, accessed on 5 July 2021) [74].

### 4.6. Cis-Acting Element Analysis

Sequences starting at 2000 bp upstream of the start codon in PtrUSPs were downloaded from Phytozome. Cis-acting elements in sequences were extracted using PlantCARE (http://bioinformatics.psb.ugent.be/webtools/plantcare/html/, accessed on 12 July 2021) [75], and results were visualized in TBtools.

### 4.7. Chromosome Distribution and Collinearity Analysis

PtrUSPs were mapped to the genome of *P. trichocarpa*, and the chromosomal distribution of PtrUSPs was visualized in TBtools. Tandem duplication events in PtrUSPs were analyzed by MCScanX [76]. Segmental duplication events and collinearity between PtrUSPs and homologous genes with other species (*A. thaliana*, *E. grandis*, *G. max*, and *S. lycopersicum*) were analyzed by Dual Synteny Plotter [77]. Results were visualized in TBtools. Ratios of non-synonymous substitution to synonymous substitution (Ka/Ks) for duplicate gene pairs were determined by the KaKs_Calculator [78].

### 4.8. Sample Preparation

Wild-type Pdpap used in this study were grown on 0.5× Murashige and Skoog (MS) medium containing 0.01 mg/mL 1-naphthaleneacetic acid (NAA). Two-month-old Pdpap at the same growth state were selected for subsequent experiments. In treatment groups, 50 mL of *F. oxysporum* with a spore density of 1 × 10^5^ per 1 mL was poured onto roots of Pdpap for 6, 12, 24, and 48 h [79]. Wild-type Pdpap treated with 50 mL ddH_2_O were used as controls. Three replicates were set for each treatment. Experimental materials and methods involved in this study are detailed in a previous publication from our group [80].

### 4.9. Gene Expression Analysis

Previous research analyzed the transcriptome of Pdpap infected by *F. oxysporum* in detail [80]. This study explored gene expression patterns of PdPapUSPs under *F. oxysporum* based on RNA-Seq. DEGs were identified by DESeq2 using log_2_ (fold change) ≥ 1 and adjusted *p*-value (padj) ≤ 0.05 as a criterion.

This study analyzed expression levels of PdPapUSPs by qRT-PCR to validate the RNA-Seq data. qRT-PCR was performed using the 2× SYBR Green qPCR Master Mix kit (Bimake, Shanghai, China), and the instrument was a Stratagene Mx3000P real-time PCR system (Agilent Technologies, Santa Clara, CA, USA). Primer sequences were designed by Primer5.0 [81] (Table S12). Reaction systems and amplification conditions of qRT-PCR refer to previous results from our group [80]. Three replicates were set up for each gene. *PdpapActin* and *PdPapEF1-α* were selected as internal control genes [82]. Relative expression levels of genes were calculated by the 2^−ΔΔCt^ method [83].

### 4.10. Co-Expression Network and Gene Ontology Annotation Analysis

Co-expression network analysis was performed on 46 PdPapUSPs identified from RNA-Seq by STRING [84]. Results were visualized using Cytoscape [85].

Using gene ontology (GO), PtrUSPs were annotated by agriGO v2.0 (http://systemsbiology.cau.edu.cn/agriGOv2/index.php, accessed on 18 August 2021) [86].

### 4.11. Statistical Analysis

Data involved in this study were analyzed by Statistical Package for Social Sciences (SPSS) 17.0 [87]. Data were compared by Student’s *t*-test and *p* < 0.05 was considered statistically significant [88]. Different lowercase letters in figures indicate degree of significant difference (*p* < 0.05).

## Data Availability

The datasets generated and/or analysed during the current study are available in the National Center for Biotechnology Information (NCBI) BioProject database under accession number PRJNA741264.

## Supplementary Materials

The supporting information can be downloaded at: https://www.mdpi.com/article/10.3390/ijms24065405/s1.
