# Supplementary material for: Comprehensive Analysis of Universal Stress Protein Family Genes and Their Expression in Fusarium oxysporum Response of Populus davidiana × P. alba var. pyramidalis Louche Based on the Transcriptome
Source: Int J Mol Sci. 2023 Mar 11;24(6):5405. doi: 10.3390/ijms24065405 (PMC10049587; doi:10.3390/ijms24065405)
Supplement: Supplementary file 1 [file ijms-24-05405-s001.zip › Table S1 Physicochemical properties of PtrUSPs.pdf]

Table S1. Physicochemical properties of PtrUSPs

Name in this paper	Gene ID	Locus tag	Number of amino acids	Molecular weight (kDa)	Theoretical pI	Aliphatic index	Grand average of hydropathicity (GRAVY)	Formula	Total number of atoms	Instability index	stability
PtrUSP1	112326636	POPTR_001G409100v3	238	26.550	6.30	77.82	-0.629	$C_{1,163}H_{1,836}N_{340}O_{357}S_{8}$	3,704	50.68	unstable
PtrUSP2	18095671	POPTR_001G414800v3	242	27.062	6.30	76.53	-0.676	$C_{1,183}H_{1,868}N_{348}O_{365}S_{8}$	3,772	53.19	unstable
PtrUSP3	7466474	POPTR_002G084600v3	240	26.519	9.13	79.62	-0.247	$C_{1,157}H_{1,843}N_{331}O_{351}S_{16}$	3,698	54.58	unstable
PtrUSP4	7461816	POPTR_002G104700v3	179	18.960	6.08	98.04	0.129	$C_{834}H_{1,358}N_{230}O_{252}S_{10}$	2,684	51.67	unstable
PtrUSP5	7481410	POPTR_002G193800v3	163	17.721	6.65	99.75	0.148	$C_{792}H_{1,276}N_{212}O_{231}S_{8}$	2,519	34.21	stable
PtrUSP6	7481397	POPTR_002G196700v3	179	19.760	6.12	92.40	-0.077	$C_{871}H_{1,391}N_{237}O_{268}S_{9}$	2,776	30.14	stable
PtrUSP7	7487779	POPTR_002G205300v3	221	23.911	7.69	94.30	0.047	$C_{1,059}H_{1,697}N_{293}O_{314}S_{11}$	3,374	38.06	stable
PtrUSP8	7494517	POPTR_004G075400v3	162	17.658	6.60	98.02	-0.149	$C_{786}H_{1,274}N_{214}O_{238}S_{4}$	2,516	23.22	stable
PtrUSP9	7469997	POPTR_004G156100v3	191	21.260	7.72	106.60	-0.114	$C_{940}H_{1,549}N_{269}O_{272}S_{9}$	3,039	34.09	stable
PtrUSP10	7461225	POPTR_004G156200v3	195	21.874	7.05	102.92	0.031	$C_{974}H_{1,567}N_{271}O_{280}S_{10}$	3,102	39.29	stable
PtrUSP11	112327648	POPTR_005G015200v3	170	18.938	7.60	87.65	-0.150	$C_{835}H_{1,340}N_{230}O_{245}S_{13}$	2,663	28.19	stable
PtrUSP12	18098600	POPTR_005G018900v3	170	18.952	7.58	89.35	-0.119	$C_{836}H_{1,342}N_{230}O_{245}S_{13}$	2,666	28.02	stable
PtrUSP13	7469151	POPTR_005G177100v3	218	23.719	9.10	83.58	-0.166	$C_{1,027}H_{1,668}N_{294}O_{319}S_{15}$	3,323	56.91	unstable
PtrUSP14	7454965	POPTR_006G092700v3	157	17.452	6.28	104.90	-0.149	$C_{776}H_{1,260}N_{210}O_{233}S_{6}$	2,485	32.08	stable
PtrUSP15	112327999	POPTR_006G225300v3	709	78.482	8.77	78.38	-0.482	$C_{3,423}H_{5,515}N_{995}O_{1,057}S_{30}$	11,020	43.03	unstable
PtrUSP16	18100796	POPTR_006G279500v3	774	85.941	8.50	78.95	-0.428	$C_{3,769}H_{6,037}N_{1,071}O_{1,160}S_{32}$	12,069	50.39	unstable
PtrUSP17	7483898	POPTR_008G109000v3	237	26.515	9.79	89.62	-0.470	$C_{1,156}H_{1,928}N_{346}O_{341}S_{12}$	3,783	41.96	unstable
PtrUSP18	7488363	POPTR_008G121800v3	167	18.052	8.85	101.56	0.142	$C_{791}H_{1,309}N_{217}O_{234}S_{14}$	2,565	36.16	stable
PtrUSP19	7488364	POPTR_008G121900v3	161	17.521	5.58	101.12	-0.038	$C_{776}H_{1,255}N_{205}O_{240}S_{7}$	2,483	29.56	stable
PtrUSP20	18101803	POPTR_008G221300v3	216	23.666	8.92	97.45	0.188	$C_{1,058}H_{1,690}N_{294}O_{291}S_{15}$	3,348	34.50	stable
PtrUSP21	112328416	POPTR_008G226400v3	216	23.638	8.91	97.45	0.191	$C_{1,058}H_{1,690}N_{292}O_{291}S_{15}$	3,346	34.11	stable
PtrUSP22	7464025	POPTR_009G117500v3	177	19.748	5.66	101.86	-0.125	$C_{868}H_{1,407}N_{249}O_{260}S_{8}$	2,792	36.77	stable
PtrUSP23	7475582	POPTR_010G123200v3	161	17.615	5.56	101.12	-0.063	$C_{780}H_{1,261}N_{209}O_{239}S_{7}$	2,496	33.03	stable
PtrUSP24	7475583	POPTR_010G123300v3	169	18.323	5.22	104.38	0.189	$C_{808}H_{1,322}N_{206}O_{249}S_{13}$	2,598	33.70	stable
PtrUSP25	7475584	POPTR_010G123400v3	173	18.779	6.08	93.58	-0.069	$C_{827}H_{1,331}N_{225}O_{254}S_{9}$	2,646	37.10	stable
PtrUSP26	7482255	POPTR_010G140200v3	236	26.235	9.84	92.12	-0.403	$C_{1,151}H_{1,924}N_{342}O_{331}S_{12}$	3,760	43.87	unstable
PtrUSP27	7468095	POPTR_010G144100v3	164	18.099	5.39	100.30	-0.074	$C_{806}H_{1,294}N_{212}O_{241}S_{9}$	2,562	22.91	stable
PtrUSP28	7495284	POPTR_011G039800v3	241	26.433	5.21	75.19	-0.512	$C_{1,145}H_{1,817}N_{329}O_{370}S_{10}$	3,671	49.80	unstable
PtrUSP29	112323402	POPTR_011G125500v3	256	28.037	5.45	79.22	-0.579	$C_{1,220}H_{1,918}N_{356}O_{390}S_{7}$	3,891	40.42	unstable
PtrUSP30	7487084	POPTR_012G059100v3	213	23.564	9.80	99.86	0.040	$C_{1,054}H_{1,708}N_{298}O_{293}S_{10}$	3,363	42.06	unstable
PtrUSP31	7458052	POPTR_012G084700v3	234	26.515	9.20	93.68	-0.311	$C_{1,178}H_{1,908}N_{332}O_{340}S_{11}$	3,769	50.91	unstable
PtrUSP32	7481701	POPTR_013G009800v3	171	18.855	6.51	94.62	-0.143	$C_{816}H_{1,339}N_{229}O_{253}S_{14}$	2,651	31.51	stable
PtrUSP33	18104452	POPTR_013G112300v3	172	19.006	8.59	106.57	0.123	$C_{853}H_{1,384}N_{222}O_{244}S_{11}$	2,714	42.46	unstable
PtrUSP34	7494451	POPTR_013G150200v3	252	27.487	5.14	81.11	-0.499	$C_{1,188}H_{1,900}N_{342}O_{387}S_{10}$	3,827	43.92	unstable

PtrUSP35	18109283	POPTR_014G122000v3	164	18.152	6.65	93.72	0.012	$C_{800}H_{1,285}N_{217}O_{239}S_{12}$	2,553	29.89	stable
PtrUSP36	7491306	POPTR_014G130100v3	162	17.361	7.04	97.41	0.225	$C_{779}H_{1,239}N_{207}O_{227}S_{7}$	2,459	22.86	stable
PtrUSP37	18105772	POPTR_015G060700v3	227	25.066	10.76	75.55	-0.456	$C_{1,085}H_{1,791}N_{343}O_{313}S_{13}$	3,545	52.51	unstable
PtrUSP38	7453799	POPTR_015G083100v3	239	27.009	9.34	92.09	-0.305	$C_{1,190}H_{1,938}N_{340}O_{349}S_{13}$	3,830	56.69	unstable
PtrUSP39	7488056	POPTR_016G064000v3	213	23.551	5.99	94.79	-0.207	$C_{1,036}H_{1,692}N_{284}O_{315}S_{12}$	3,339	38.48	stable
PtrUSP40	7486537	POPTR_016G104600v3	161	17.762	5.20	102.24	-0.180	$C_{783}H_{1,270}N_{214}O_{241}S_{7}$	2,515	27.64	stable
PtrUSP41	7496651	POPTR_017G071700v3	176	19.906	5.97	75.80	-0.426	$C_{876}H_{1,373}N_{249}O_{262}S_{10}$	2,770	40.01	unstable
PtrUSP42	7463713	POPTR_018G061600v3	760	84.311	8.30	78.47	-0.533	$C_{3,673}H_{5,884}N_{1,072}O_{1,147}S_{28}$	11,804	42.34	unstable
PtrUSP43	7458850	POPTR_019G119400v3	252	27.517	4.84	81.94	-0.504	$C_{1,191}H_{1,894}N_{342}O_{387}S_{10}$	3,824	50.21	unstable
PtrUSP44	7460863	POPTR_T024200v3	256	28.051	5.45	79.22	-0.579	$C_{1,221}H_{1,920}N_{356}O_{390}S_{7}$	3,894	39.67	stable
PtrUSP45	7496605	POPTR_T059500v3	161	17.617	7.81	96.21	-0.245	$C_{787}H_{1,271}N_{215}O_{236}S_{3}$	2,512	27.86	stable
PtrUSP46	112325879	POPTR_T120500v3	161	17.444	6.59	98.63	-0.106	$C_{776}H_{1,256}N_{212}O_{235}S_{4}$	2,483	23.54	stable
