# Supplementary material for: Comprehensive Analysis of Universal Stress Protein Family Genes and Their Expression in Fusarium oxysporum Response of Populus davidiana × P. alba var. pyramidalis Louche Based on the Transcriptome
Source: Int J Mol Sci. 2023 Mar 11;24(6):5405. doi: 10.3390/ijms24065405 (PMC10049587; doi:10.3390/ijms24065405)
Supplement: Supplementary file 1 [file ijms-24-05405-s001.zip › Table S11 Gene ontology analysis.pdf]

Table S11. Gene ontology analysis

GO term	Ontology	Description	Number in input list	Number in BG/Ref	p-value	FDR
GO:0006950	Biological Process	response to stress	45	730	9.9×10^{-65}	3×10^{-64}
GO:0050896	Biological Process	response to stimulus	45	910	1.5×10^{-60}	2.3×10^{-60}
