# Supplementary material for: Comprehensive Analysis of Universal Stress Protein Family Genes and Their Expression in Fusarium oxysporum Response of Populus davidiana × P. alba var. pyramidalis Louche Based on the Transcriptome
Source: Int J Mol Sci. 2023 Mar 11;24(6):5405. doi: 10.3390/ijms24065405 (PMC10049587; doi:10.3390/ijms24065405)
Supplement: Supplementary file 1 [file ijms-24-05405-s001.zip › Table S12 Primer Sequences of qRT-PCR.pdf]

Table S12. Primer Sequences of qRT-PCR

Gene	Forward Primers (5'-3')	Reverse Primers (5'-3')
Pdpapactin	GCTGAGAGATTCCGTTGCCCTG	GGCGGTGATCTCCTTGCTCATT
PdpapEF1-α	TGGGTCGTGTTGAAACTGGTGT	GGCAGGATCGTCCTTGGAGTTC
PdpapUSP1	GATTTCGATGCTTTCACGGC	TCTCATCGCTCCCTCTTATCG
PdpapUSP2	GATTTCGATGCTTTCACGGC	TCTCATCGCTCCCTCTTATCG
PdpapUSP3	CCGACCTGAGAGCTGTTTG	TTCCTTACACCAATAGCCCAG
PdpapUSP4	GAACATGCCTTGGAGATTTGC	ACACTTCCCAGAAACATCCTG
PdpapUSP5	CCAAAACTCCCGCTACCTC	ATGACTTCCTACAACGAGCAC
PdpapUSP6	AAAGTAATTGGGAGTGGAGAGG	AAGAGGTGGATGGGAGAGTAG
PdpapUSP7	CCTGGGATTGCTGAAGTTTTG	TCACACTACCTAAAACTGCCC
PdpapUSP8	ACAGTTTCAAGGACCAAGGG	ACACTCCCAAGCAACACC
PdpapUSP9	CACCATTCATCTTGTTCATGCC	GATTAAGCCTCTGCCTCTAGTG
PdpapUSP10	CACCCTCCATCTTGTCCATG	ATTACCTTTCCAGCATCCCC
PdpapUSP11	TGCTCTTTCATGGTGTCTCG	TCGTCTCCACCTTAACATCTTG
PdpapUSP12	GCTCTTTCATGGTGTCTCGAG	CATGCTCTATTCTCGTCTCCAC
PdpapUSP13	TTGCTAGTTCTCTTGGGTCAC	TTCCTTACACCGACAGTCAAG
PdpapUSP14	GCTTGACACCATTTCACGAC	TCACACTCCCCAGAAGTATCC
PdpapUSP15	CTCAAAATACAGGGACATTCGC	ACCTCGTCAACCTTCCATTG
PdpapUSP16	TCAGTTGGCGATACAGAATCC	ATGCTGGAAATATGCGATCAATG
PdpapUSP17	GTGTCAGTTGAAGAGACCTGAG	TCTGCCCCAAAACTAGAAGTG
PdpapUSP18	CATATGTCCACTCTCAGCCAC	CCTCAGTAATTGCCTCCACAG
PdpapUSP19	TGTTGCAGAGACAGTAACGG	TGACACTTCCCAGAAAAGCC
PdpapUSP20	GCTAGAAATGTCATGTGCGATG	ACAATCAACACAGAGCAGGG
PdpapUSP21	CTTTGTTCCATTTGGTCAGGAC	ACATTTTGGGCTCTTTTCTTGG
PdpapUSP22	CTTGTCCATGCTGTCTCTAGTG	ATTATCTTACCAGCATCCCCTTC
PdpapUSP23	CTGGGTTATCTCTATGCTTCCAC	GTCATTATCTCTACATCGACCCC
PdpapUSP24	GCTGTGGATATCTGTGATAGTCG	AGGGTTCCATTAACGAGTGTG
PdpapUSP25	CTGAGACATTAACAGAGGTGGG	ACTGCTCACACTTCCTTGG
PdpapUSP26	GTGTCAGTTGAAGAGACCTGAG	CTGCCCCAGAACTAGAAGTG
PdpapUSP27	CCTCGTGAAAAGATATGTGAAGC	CAACAGTAATAGGGCAGGATCC
PdpapUSP28	AATGATATAGCACAGCCTCTTGT	CAAACCCTCTACTCCCCATTAC
PdpapUSP29	GTTGAGAGGTTAGGGTTGAGTG	CCACGACTGGACATACACAG
PdpapUSP30	GCACCAGACATTTCAATCAGC	CTCTTTGACTTCCTCGATCTCC
PdpapUSP31	AGCTCATTCCCGTTCTCAAC	CACAAATTCTCCTTTCGCCAG
PdpapUSP32	AAGGGATCTCATTTGCCAGG	ATCAGAACAGGGCACTTCAC
PdpapUSP33	CTCTCCCACCCTAGATTTGATC	ATGTGAGTTCATAAGGCTCCC
PdpapUSP34	ACAAATCTGAATCTGACCTCCC	CTGCCCATTTGACTGCATATG
PdpapUSP35	GAGTTATTGGGAGTGGAGAGG	GCTTGGCACAATGTTCACTC
PdpapUSP36	TTCTTTGCGAGGCTGTAGAC	AGGCCTCTTCACAATCATGAC
PdpapUSP37	ATGAGACGCTTCACTCCATG	TTCTTTACCCTCATGCACCG
PdpapUSP38	GATGGTAAGGATGGGATAGAGC	CCATCACCATGTTCCTACAAACT
PdpapUSP39	CAGGTTGATCTCCTTGTTCTAGG	GGTCTCCTTAGTGATCTCCTTTG
PdpapUSP40	GCTTGACACCATTTCACGAC	TGCTTCCCATGACCAAAGAG

PdpapUSP41	CATGAAGCATAGGGATAGGACC	TGGCTGGACTCGTTTTACTTC
PdpapUSP42	CCACTTCTGTAGCCATCGAC	ACCATCCTGACCATTAGCAAG
PdpapUSP43	CCAATTTGCTGGCTAAACCAT	CCTCTACTTCCCATTATCACAGC
PdpapUSP44	GTTGAGAGGTTAGGGTTGAGTG	CCACGACTGGACATACACAG
PdpapUSP45	ATCCAAGGGAGAAGCTGATTG	GTGACTGGACATGAAGCATTTG
PdpapUSP46	GGAGATCCAAGGGAGAAGTTG	TGACTGGACATGGAGCATTTG
