# Supplementary material for: Comprehensive Analysis of Universal Stress Protein Family Genes and Their Expression in Fusarium oxysporum Response of Populus davidiana × P. alba var. pyramidalis Louche Based on the Transcriptome
Source: Int J Mol Sci. 2023 Mar 11;24(6):5405. doi: 10.3390/ijms24065405 (PMC10049587; doi:10.3390/ijms24065405)
Supplement: Supplementary file 1 [file ijms-24-05405-s001.zip › Table S2 Annotations of PtrUSP sequence motifs.pdf]

Table S2. Annotations of PtrUSP sequence motifs

Name	Sequence	Description of Pfam	Distribution in PtrUSPs
Motif 1	VEEGDPKEVJCDAVEKLKLSALVMGSRGYGA	Universal stress protein family	PtrUSP1, PtrUSP2, PtrUSP4, PtrUSP5, PtrUSP6, PtrUSP7, PtrUSP8, PtrUSP9, PtrUSP10, PtrUSP11, PtrUSP12, PtrUSP14, PtrUSP18, PtrUSP19, PtrUSP20, PtrUSP21, PtrUSP22, PtrUSP23, PtrUSP24, PtrUSP25, PtrUSP27, PtrUSP28, PtrUSP29, PtrUSP32, PtrUSP33, PtrUSP34, PtrUSP35, PtrUSP36, PtrUSP39, PtrUSP40, PtrUSP41, PtrUSP43, PtrUSP44, PtrUSP45, PtrUSP46
Motif 2	IMVAVDESEESKYALKWALDNL	Universal stress protein family	PtrUSP1, PtrUSP2, PtrUSP3, PtrUSP4, PtrUSP5, PtrUSP6, PtrUSP7, PtrUSP8, PtrUSP9, PtrUSP10, PtrUSP11, PtrUSP12, PtrUSP13, PtrUSP14, PtrUSP15, PtrUSP16, PtrUSP17, PtrUSP18, PtrUSP19, PtrUSP20, PtrUSP21, PtrUSP22, PtrUSP23, PtrUSP24, PtrUSP25, PtrUSP26, PtrUSP27, PtrUSP28, PtrUSP29, PtrUSP30, PtrUSP31, PtrUSP32, PtrUSP33, PtrUSP34, PtrUSP35, PtrUSP36, PtrUSP37, PtrUSP38, PtrUSP39, PtrUSP40, PtrUSP41, PtrUSP42, PtrUSP43, PtrUSP44, PtrUSP45, PtrUSP46
Motif 3	LGSVSDYCVHNAKCPV	Universal stress protein family	PtrUSP1, PtrUSP2, PtrUSP4, PtrUSP5, PtrUSP6, PtrUSP7, PtrUSP8, PtrUSP9, PtrUSP10, PtrUSP11, PtrUSP12, PtrUSP14, PtrUSP17, PtrUSP18, PtrUSP19, PtrUSP20, PtrUSP21, PtrUSP22, PtrUSP23, PtrUSP24, PtrUSP25, PtrUSP26, PtrUSP27, PtrUSP28, PtrUSP29, PtrUSP32, PtrUSP33, PtrUSP34, PtrUSP35, PtrUSP36, PtrUSP37, PtrUSP39, PtrUSP40, PtrUSP41, PtrUSP43, PtrUSP44, PtrUSP45, PtrUSP46
Motif 4	RPGDAVILLHVSPTSVLYGADWGPLPLSTPTQSQLDLLNNESK	Universal stress protein family	PtrUSP1, PtrUSP2, PtrUSP28, PtrUSP29, PtrUSP31, PtrUSP34, PtrUSP38, PtrUSP43, PtrUSP44
Motif 5	GTAGPGSADLJPSMZEDNKKIALRVJEKAKEICAEKGVSVEVV	None	PtrUSP4, PtrUSP5, PtrUSP7, PtrUSP11, PtrUSP12, PtrUSP18, PtrUSP19, PtrUSP20, PtrUSP21, PtrUSP23, PtrUSP24, PtrUSP25, PtrUSP32, PtrUSP33, PtrUSP36
Motif 6	TALLFLHQTKPEPLVHRDLKPANILLDRNYVSKISDVGLA	Protein kinase domain	PtrUSP15, PtrUSP16, PtrUSP42

	RLVPPSVADSVTQYHMTSAAGTFCYIDPEYQQTGMLTTRSDIYSLGIMFLQIITAKPPMG		
Motif 7	HNDVRYRRYTIEEIEEATDKFSPSNKIGEGGYGPVYKGKLDHTPVAIKALRPDAAQGKKQFQQEVEVLSCIRHPHMVLLLGACPEYGVLVYEFMDNGSLE	Protein tyrosine and serine/threonine kinase	PtrUSP15, PtrUSP16, PtrUSP42
Motif 8	QVQPPNADHTRKQLFEETGSPLIPLAEFREPNFSKQYGLTYDPEVLDILDTVSRTKG	None	PtrUSP8, PtrUSP14, PtrUSP27, PtrUSP40, PtrUSP45, PtrUSP46
Motif 9	LEDDFDAFTASKAADJARPLKEAQIPYKIHI	None	PtrUSP1, PtrUSP2, PtrUSP28, PtrUSP29, PtrUSP34, PtrUSP43, PtrUSP44
Motif 10	ADHLEAEMRRLRLELKQTMEMYSTACKEALSAKQKASELNQWKIDEVRKFEEAKLAGEAALAIAELEKAKCKVAIEAAEKSQKLAELEAQKRKHAEMKAE	PAXX, PAralog of XRCC4 and XLF, also called C9orf142	PtrUSP15, PtrUSP16, PtrUSP42
Motif 11	QQQQQNPVDPDQPQLPTIKIHHPPSPPRHPH	None	PtrUSP1, PtrUSP2, PtrUSP29, PtrUSP44
Motif 12	SNSGDTLVJLHVQPPS	None	PtrUSP3, PtrUSP4, PtrUSP5, PtrUSP6, PtrUSP7, PtrUSP11, PtrUSP12, PtrUSP13, PtrUSP15, PtrUSP16, PtrUSP17, PtrUSP18, PtrUSP19, PtrUSP20, PtrUSP21, PtrUSP23, PtrUSP24, PtrUSP25, PtrUSP26, PtrUSP28, PtrUSP30, PtrUSP32, PtrUSP33, PtrUSP35, PtrUSP36, PtrUSP37, PtrUSP39, PtrUSP41, PtrUSP42
Motif 13	RRKSKKHGGYLITTKRHKDFWL	None	PtrUSP3, PtrUSP13, PtrUSP17, PtrUSP26, PtrUSP37
Motif 14	MAVTANLLIFFILLLTYCNIVISTCLKLEIFKSRAGFCFCWRREGELVGAKAKKTAKMAG	None	PtrUSP20, PtrUSP21
Motif 15	KNMCQLKRPEIQIEIAVVEGKEKGPLIVEEAKKQGVALLVLGQKKRSMTWRLIMMWASN	Universal stress protein family	PtrUSP17, PtrUSP26, PtrUSP37
Motif 16	TLKFKKQDVPESVKKIAPDFCSVYVITKGK	Sporulation lipoprotein YhcN/YlaJ (Spore_YhcN_YlaJ)	PtrUSP15, PtrUSP16, PtrUSP31, PtrUSP38, PtrUSP42
Motif 17	HQVGRAIERGGFADMLDQTVPDWPVEEALRFAKLALKCAELRKKDRPSLATVIVPELNRL	Protein of unknown function (DUF3012)	PtrUSP15, PtrUSP16, PtrUSP42

Motif 18	CRLADTIHLVHAVSSVQNTVVYETSQQLLEKLAVEALQVAMV	None	PtrUSP9, PtrUSP10, PtrUSP22
Motif 19	LVVKKPK	None	PtrUSP1, PtrUSP2, PtrUSP3, PtrUSP4, PtrUSP5, PtrUSP6, PtrUSP7, PtrUSP8, PtrUSP11, PtrUSP12, PtrUSP13, PtrUSP14, PtrUSP19, PtrUSP20, PtrUSP21, PtrUSP23, PtrUSP24, PtrUSP27, PtrUSP28, PtrUSP29, PtrUSP32, PtrUSP33, PtrUSP34, PtrUSP35, PtrUSP36, PtrUSP39, PtrUSP40, PtrUSP44, PtrUSP45, PtrUSP46
Motif 20	QPYRGYCARKGVTVKTVVLEDNDVAKAIAEYIPVLLIGNJVMG	None	PtrUSP15, PtrUSP16, PtrUSP31, PtrUSP38, PtrUSP42
