# Supplementary material for: Comprehensive Analysis of Universal Stress Protein Family Genes and Their Expression in Fusarium oxysporum Response of Populus davidiana × P. alba var. pyramidalis Louche Based on the Transcriptome
Source: Int J Mol Sci. 2023 Mar 11;24(6):5405. doi: 10.3390/ijms24065405 (PMC10049587; doi:10.3390/ijms24065405)
Supplement: Supplementary file 1 [file ijms-24-05405-s001.zip › Table S3 Predition of the Sencondary Structure in PtrUSPs.pdf]

Table S3. Predition of the Sencondary Structure in PtrUSPs

Name in this paper	Gene ID	Locus tag	Number of Alpha helix	The percent of all the sencondary structures	Number of Extended strand	The percent of all the sencondary structures	Number of Beta turn	The percent of all the sencondary structures	Number of Random coil	The percent of all the sencondary structures
PtrUSP1	112326636	POPTR_001G409100v3	64	26.89%	42	17.65%	13	5.46%	119	50.00%
PtrUSP2	18095671	POPTR_001G414800v3	76	31.40%	34	14.05%	18	7.44%	114	47.11%
PtrUSP3	7466474	POPTR_002G084600v3	85	35.42%	48	20.00%	17	7.08%	90	37.50%
PtrUSP4	7461816	POPTR_002G104700v3	75	41.90%	31	17.32%	8	4.47%	65	36.31%
PtrUSP5	7481410	POPTR_002G193800v3	70	42.94%	35	21.47%	9	5.52%	49	30.06%
PtrUSP6	7481397	POPTR_002G196700v3	74	41.34%	28	15.64%	9	5.03%	68	37.99%
PtrUSP7	7487779	POPTR_002G205300v3	88	39.82%	48	21.72%	14	6.33%	71	32.13%
PtrUSP8	7494517	POPTR_004G075400v3	59	36.42%	33	20.37%	8	4.94%	62	38.27%
PtrUSP9	7469997	POPTR_004G156100v3	95	49.74%	37	19.37%	10	5.24%	49	25.65%
PtrUSP10	7461225	POPTR_004G156200v3	102	52.31%	38	19.49%	11	5.64%	44	22.56%
PtrUSP11	112327648	POPTR_005G015200v3	73	42.94%	30	17.65%	11	6.47%	56	32.94%
PtrUSP12	18098600	POPTR_005G018900v3	75	44.12%	31	18.24%	8	4.71%	56	32.94%
PtrUSP13	7469151	POPTR_005G177100v3	69	31.65%	47	21.56%	13	5.96%	89	40.83%
PtrUSP14	7454965	POPTR_006G092700v3	61	38.85%	35	22.29%	8	5.10%	53	33.76%
PtrUSP15	112327999	POPTR_006G225300v3	46	31.51%	24	16.44%	10	6.85%	66	45.21%
PtrUSP16	18100796	POPTR_006G279500v3	286	36.95%	82	10.59%	32	4.13%	374	48.32%
PtrUSP17	7483898	POPTR_008G109000v3	51	21.52%	48	20.25%	14	5.91%	124	52.32%
PtrUSP18	7488363	POPTR_008G121800v3	68	40.72%	33	19.76%	13	7.78%	53	31.74%
PtrUSP19	7488364	POPTR_008G121900v3	70	43.48%	32	19.88%	10	6.21%	49	30.43%
PtrUSP20	18101803	POPTR_008G221300v3	101	46.76%	53	24.54%	18	8.33%	44	20.37%
PtrUSP21	112328416	POPTR_008G226400v3	94	43.52%	55	25.46%	17	7.87%	50	23.15%
PtrUSP22	7464025	POPTR_009G117500v3	96	54.24%	29	16.38%	5	2.82%	47	26.55%
PtrUSP23	7475582	POPTR_010G123200v3	66	40.99%	29	18.01%	10	6.21%	56	34.78%
PtrUSP24	7475583	POPTR_010G123300v3	71	42.01%	31	18.34%	12	7.10%	55	32.54%
PtrUSP25	7475584	POPTR_010G123400v3	76	43.93%	30	17.34%	11	6.36%	56	32.37%
PtrUSP26	7482255	POPTR_010G140200v3	53	22.46%	45	19.07%	11	4.66%	127	53.81%
PtrUSP27	7468095	POPTR_010G144100v3	70	42.68%	35	21.34%	12	7.32%	47	28.66%
PtrUSP28	7495284	POPTR_011G039800v3	87	36.10%	47	19.50%	9	3.73%	98	40.66%
PtrUSP29	112323402	POPTR_011G125500v3	72	28.12%	40	15.62%	14	5.47%	130	50.78%
PtrUSP30	7487084	POPTR_012G059100v3	56	26.29%	52	24.41%	9	4.23%	96	45.07%
PtrUSP31	7458052	POPTR_012G084700v3	78	33.33%	38	16.24%	11	4.70%	107	45.73%
PtrUSP32	7481701	POPTR_013G009800v3	74	43.27%	31	18.13%	8	4.68%	58	33.92%
PtrUSP33	18104452	POPTR_013G112300v3	70	40.70%	33	19.19%	11	6.40%	58	33.72%
PtrUSP34	7494451	POPTR_013G150200v3	75	29.76%	42	16.67%	11	4.37%	124	49.21%
PtrUSP35	18109283	POPTR_014G122000v3	72	43.90%	35	21.34%	9	5.49%	48	29.27%
PtrUSP36	7491306	POPTR_014G130100v3	66	40.74%	34	20.99%	8	4.94%	54	33.33%

PtrUSP37	18105772	POPTR_015G060700v3	57	25.11%	54	23.79%	11	4.85%	105	46.26%
PtrUSP38	7453799	POPTR_015G083100v3	69	28.87%	44	18.41%	7	2.93%	119	49.79%
PtrUSP39	7488056	POPTR_016G064000v3	97	45.54%	34	15.96%	12	5.63%	70	32.86%
PtrUSP40	7486537	POPTR_016G104600v3	67	41.61%	34	21.12%	6	3.73%	54	33.54%
PtrUSP41	7496651	POPTR_017G071700v3	70	39.77%	27	15.34%	8	4.55%	71	40.34%
PtrUSP42	7463713	POPTR_018G061600v3	286	37.63%	75	9.87%	37	4.87%	362	47.63%
PtrUSP43	7458850	POPTR_019G119400v3	68	26.98%	52	20.63%	17	6.75%	115	45.63%
PtrUSP44	7460863	POPTR_T024200v3	70	27.34%	41	16.02%	12	4.69%	133	51.95%
PtrUSP45	7496605	POPTR_T059500v3	61	37.89%	34	21.12%	12	7.45%	54	33.54%
PtrUSP46	112325879	POPTR_T120500v3	60	37.27%	33	20.50%	9	5.59%	59	36.65%
