# Supplementary material for: Comprehensive Analysis of Universal Stress Protein Family Genes and Their Expression in Fusarium oxysporum Response of Populus davidiana × P. alba var. pyramidalis Louche Based on the Transcriptome
Source: Int J Mol Sci. 2023 Mar 11;24(6):5405. doi: 10.3390/ijms24065405 (PMC10049587; doi:10.3390/ijms24065405)
Supplement: Supplementary file 1 [file ijms-24-05405-s001.zip › Table S4 Predition of the protein structure homology-modelling in PtrUSPs.pdf]

Table S4. Predition of the protein structure homology-modelling in PtrUSPs

Name in this paper	Gene ID	Locus tag	Template	Sequence Identity	Description	Homology-modelling
PtrUSP1	112326636	POPTR_001G409100v3	1mjh.1.A	23.60%	Structure-based assignment of the biochemical function of hypothetical protein MJ0577: A test case of structural genomics.	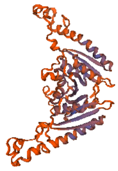
PtrUSP2	18095671	POPTR_001G414800v3	1mjh.1.A	23.13%	Structure-based assignment of the biochemical function of hypothetical protein MJ0577: A test case of structural genomics.	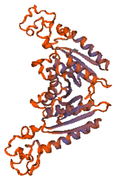
PtrUSP3	7466474	POPTR_002G084600v3	1mjh.1.A	13.91%	Structure-based assignment of the biochemical function of hypothetical protein MJ0577: A test case of structural genomics.	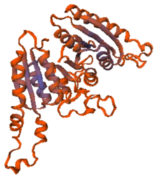
PtrUSP4	7461816	POPTR_002G104700v3	1mjh.1.A	22.98%	Structure-based assignment of the biochemical function of hypothetical protein MJ0577: A test case of structural genomics.	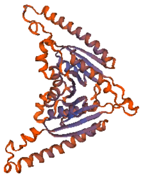
PtrUSP5	7481410	POPTR_002G193800v3	1mjh.1.A	19.74%	Structure-based assignment of the biochemical function of hypothetical protein MJ0577: A test case of structural genomics.	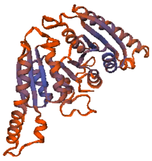
PtrUSP6	7481397	POPTR_002G196700v3	2gm3.1.A	28.21%	Crystal Structure of an Universal Stress Protein Family Protein from Arabidopsis Thaliana At3g01520 with AMP Bound.	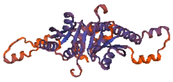

PtrUSP7	7487779	POPTR_002G205300v3	3hgm.2.A	23.61%	Universal Stress Protein TeaD; Universal Stress Protein TeaD from the TRAP transporter TeaABC of Halomonas elongata.	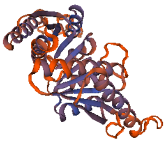
PtrUSP8	7494517	POPTR_004G075400v3	1mjh.1.A	23.72%	Structure-based assignment of the biochemical function of hypothetical protein MJ0577: A test case of structural genomics.	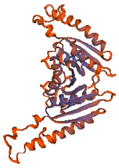
PtrUSP9	7469997	POPTR_004G156100v3	2gm3.1.A	31.40%	Crystal Structure of an Universal Stress Protein Family Protein from Arabidopsis Thaliana At3g01520 with AMP Bound	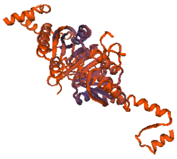
PtrUSP10	7461225	POPTR_004G156200v3	1mjh.1.A	18.80%	Structure-based assignment of the biochemical function of hypothetical protein MJ0577: A test case of structural genomics.	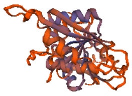
PtrUSP11	112327648	POPTR_005G015200v3	1mjh.1.A	27.45%	Structure-based assignment of the biochemical function of hypothetical protein MJ0577: A test case of structural genomics	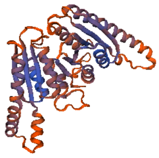
PtrUSP12	18098600	POPTR_005G018900v3	2gm3.1.A	28.21%	Crystal Structure of an Universal Stress Protein Family Protein from Arabidopsis Thaliana At3g01520 with AMP Bound.	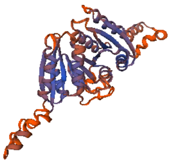
PtrUSP13	7469151	POPTR_005G177100v3	2dum.1.A	10.64%	Hypothetical protein PH0823; Crystal structure of hypothetical protein, PH0823.	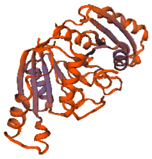

PtrUSP14	7454965	POPTR_006G092700v3	1mjh.1.A	24.18%	Structure-based assignment of the biochemical function of hypothetical protein MJ0577: A test case of structural genomics.	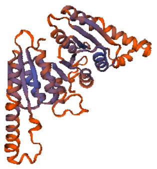
PtrUSP15	112327999	POPTR_006G225300v3	3hgm.1.A	15.00%	Universal Stress Protein TeaD. Universal Stress Protein TeaD from the TRAP transporter TeaABC of Halomonas elongata.	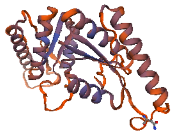
PtrUSP16	18100796	POPTR_006G279500v3	6bfn.1.A	36.14%	Interleukin-1 receptor-associated kinase 1 Crystal structure of human IRAK1.	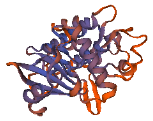
PtrUSP17	7483898	POPTR_008G109000v3	1mjh.1.A	21.28%	Structure-based assignment of the biochemical function of hypothetical protein MJ0577: A test case of structural genomics.	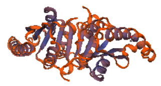
PtrUSP18	7488363	POPTR_008G121800v3	2gm3.1.A	29.87%	Crystal Structure of an Universal Stress Protein Family Protein from Arabidopsis Thaliana At3g01520 with AMP Bound.	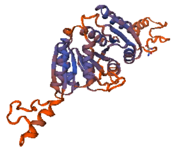
PtrUSP19	7488364	POPTR_008G121900v3	2gm3.1.A	31.17%	Crystal Structure of an Universal Stress Protein Family Protein from Arabidopsis Thaliana At3g01520 with AMP Bound.	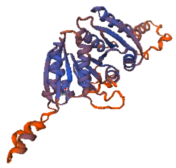
PtrUSP20	18101803	POPTR_008G221300v3	3hgm.2.A	19.44%	Universal Stress Protein TeaD; Universal Stress Protein TeaD from the TRAP transporter TeaABC of Halomonas elongata.	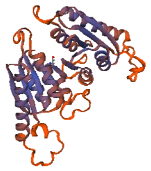

PtrUSP21	112328416	POPTR_008G226400v3	3hgm.2.A	19.44%	The crystal structure of a universal stress protein from Archaeoglobus fulgidus DSM 4304.	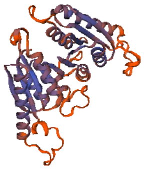
PtrUSP22	7464025	POPTR_009G117500v3	3mt0.1.A	21.25%	The crystal structure of a functionally unknown protein PA1789 from Pseudomonas aeruginosa PAO1.	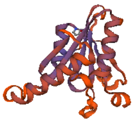
PtrUSP23	7475582	POPTR_010G123200v3	2gm3.1.A	33.77%	Crystal Structure of an Universal Stress Protein Family Protein from Arabidopsis Thaliana At3g01520 with AMP Bound.	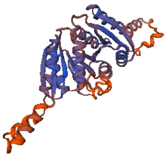
PtrUSP24	7475583	POPTR_010G123300v3	1mjh.1.A	18.13%	Structure-based assignment of the biochemical function of hypothetical protein MJ0577: A test case of structural genomics	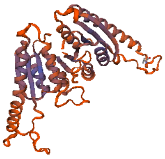
PtrUSP25	7475584	POPTR_010G123400v3	3hgm.2.A	25.69%	Universal Stress Protein TeaD Universal Stress Protein TeaD from the TRAP transporter TeaABC of Halomonas elongata.	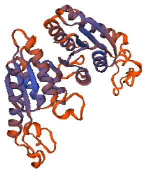
PtrUSP26	7482255	POPTR_010G140200v3	1mjh.1.A	21.43%	Structure-based assignment of the biochemical function of hypothetical protein MJ0577: A test case of structural genomics.	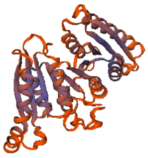
PtrUSP27	7468095	POPTR_010G144100v3	1mjh.1.A	18.87%	Structure-based assignment of the biochemical function of hypothetical protein MJ0577: A test case of structural genomics.	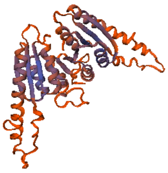

PtrUSP28	7495284	POPTR_011G039800v3	1mjh.1.A	24.36%	Structure-based assignment of the biochemical function of hypothetical protein MJ0577: A test case of structural genomics.	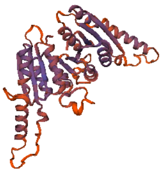
PtrUSP29	112323402	POPTR_011G125500v3	1mjh.1.A	24.53%	Structure-based assignment of the biochemical function of hypothetical protein MJ0577: A test case of structural genomics.	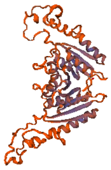
PtrUSP30	7487084	POPTR_012G059100v3	3hgm.1.A	20.30%	Universal Stress Protein TeaD; Universal Stress Protein TeaD from the TRAP transporter TeaABC of Halomonas elongata.	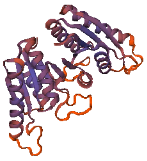
PtrUSP31	7458052	POPTR_012G084700v3	1mjh.1.A	13.77%	Structure-based assignment of the biochemical function of hypothetical protein MJ0577: A test case of structural genomics.	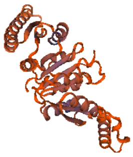
PtrUSP32	7481701	POPTR_013G009800v3	2gm3.1.A	29.30%	Crystal Structure of an Universal Stress Protein Family Protein from Arabidopsis Thaliana At3g01520 with AMP Bound.	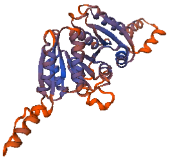
PtrUSP33	18104452	POPTR_013G112300v3	3hgm.1.A	21.53%	Universal Stress Protein TeaD; Universal Stress Protein TeaD from the TRAP transporter TeaABC of Halomonas elongata.	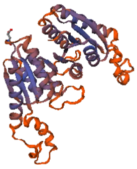
PtrUSP34	7494451	POPTR_013G150200v3	1mjh.1.A	23.75%	Structure-based assignment of the biochemical function of hypothetical protein MJ0577: A test case of structural genomics.	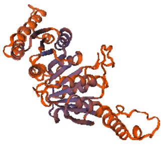

PtrUSP35	18109283	POPTR_014G122000v3	2gm3.1.A	27.27%	Crystal Structure of an Universal Stress Protein Family Protein from Arabidopsis Thaliana At3g01520 with AMP Bound.	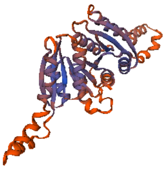
PtrUSP36	7491306	POPTR_014G130100v3	3hgm.1.A	24.31%	Universal Stress Protein TeaD; Universal Stress Protein TeaD from the TRAP transporter TeaABC of Halomonas elongata.	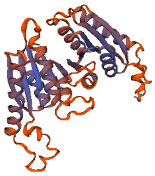
PtrUSP37	18105772	POPTR_015G060700v3	3ab7.1.B	20.71%	Crystal Structure of the Hypothetical Tandem-type Universal Stress Protein TTHA0350 from Thermus thermophilus HB8.	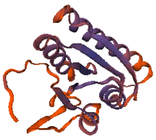
PtrUSP38	7453799	POPTR_015G083100v3	1mjh.1.A	12.75%	Structure-based assignment of the biochemical function of hypothetical protein MJ0577: A test case of structural genomics.	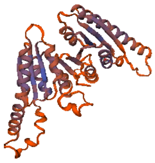
PtrUSP39	7488056	POPTR_016G064000v3	2gm3.1.A	31.65%	Crystal Structure of an Universal Stress Protein Family Protein from Arabidopsis Thaliana At3g01520 with AMP Bound	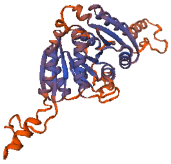
PtrUSP40	7486537	POPTR_016G104600v3	2gm3.1.A	23.18%	Crystal Structure of an Universal Stress Protein Family Protein from Arabidopsis Thaliana At3g01520 with AMP Bound.	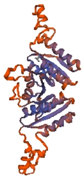
PtrUSP41	7496651	POPTR_017G071700v3	2gm3.1.A	72.99%	Crystal Structure of an Universal Stress Protein Family Protein from Arabidopsis Thaliana At3g01520 with AMP Bound.	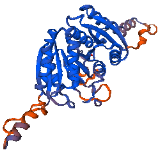

PtrUSP42	7463713	POPTR_018G061600v3	6bfn.2.A	34.04%	Interleukin-1 receptor-associated kinase 1 Crystal structure of human IRAK1	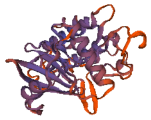
PtrUSP43	7458850	POPTR_019G119400v3	1mjh.1.A	22.01%	Structure-based assignment of the biochemical function of hypothetical protein MJ0577: A test case of structural genomics.	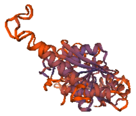
PtrUSP44	7460863	POPTR_T024200v3	1mjh.1.A	25.16%	Structure-based assignment of the biochemical function of hypothetical protein MJ0577: A test case of structural genomics.	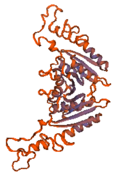
PtrUSP45	7496605	POPTR_T059500v3	1mjh.1.A	23.42%	Structure-based assignment of the biochemical function of hypothetical protein MJ0577: A test case of structural genomics.	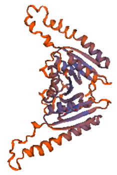
PtrUSP46	112325879	POPTR_T120500v3	1mjh.1.A	23.42%	Structure-based assignment of the biochemical function of hypothetical protein MJ0577: A test case of structural genomics.	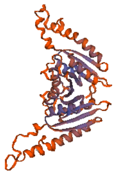
