# Supplementary material for: Comprehensive Analysis of Universal Stress Protein Family Genes and Their Expression in Fusarium oxysporum Response of Populus davidiana × P. alba var. pyramidalis Louche Based on the Transcriptome
Source: Int J Mol Sci. 2023 Mar 11;24(6):5405. doi: 10.3390/ijms24065405 (PMC10049587; doi:10.3390/ijms24065405)
Supplement: Supplementary file 1 [file ijms-24-05405-s001.zip › Table S5 Topological heterogeneity model prediction of PtrUSPs.pdf]

Table S5. Topological heterogeneity model prediction of PtrUSPs

Name	Gene ID	Locus tag	Number of N-glycosylation sites	Signal peptides	Topological heterogeneity model
PtrMFS1	112326636	POPTR_001G409100v3	1	No	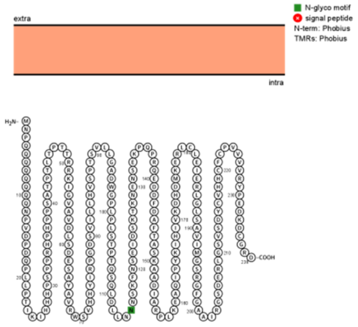
PtrMFS2	18095671	POPTR_001G414800v3	1	No	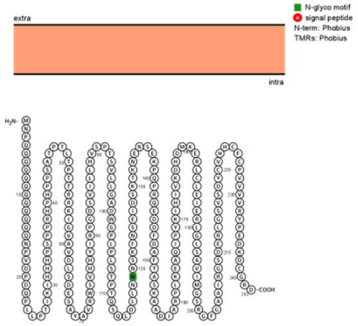

PtrMFS3	7466474	POPTR_002G084600v3	0	No	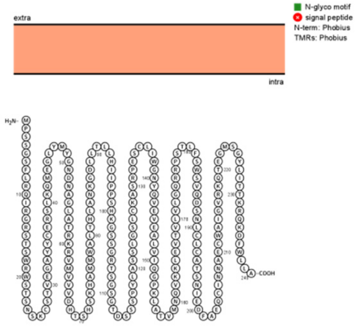
PtrMFS4	7461816	POPTR_002G104700v3	0	No	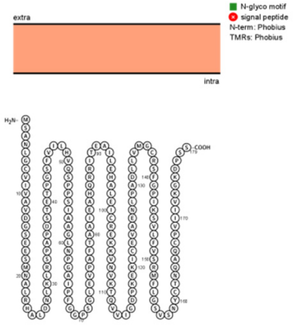
PtrMFS5	7481410	POPTR_002G193800v3	0	No	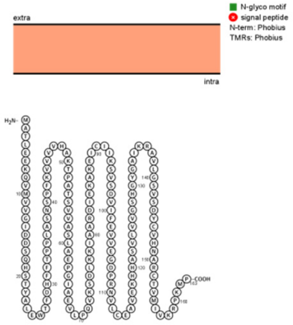

PtrMFS6	7481397	POPTR_002G196700v3	1	No	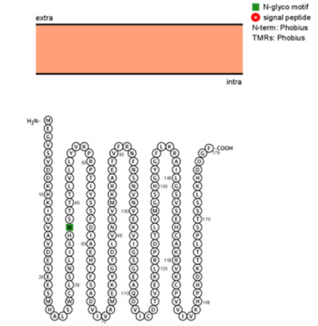
PtrMFS7	7487779	POPTR_002G205300v3	0	No	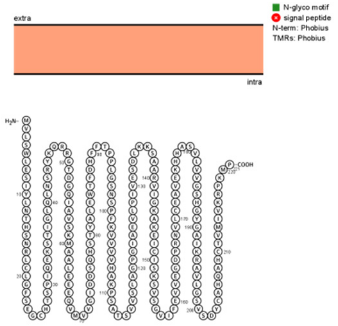
PtrMFS8	7494517	POPTR_004G075400v3	1	No	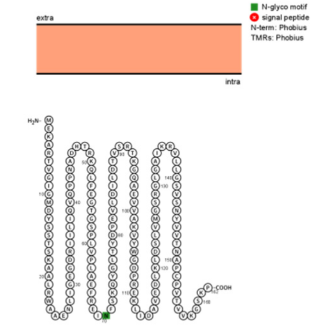

PtrMFS9	7469997	POPTR_004G156100v3	0	No	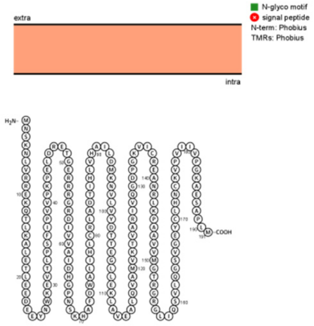
PtrMFS10	7461225	POPTR_004G156200v3	0	No	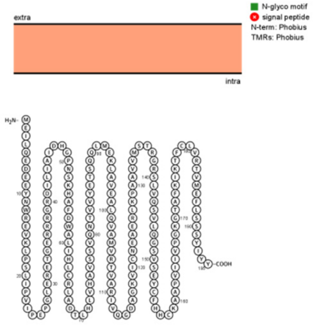
PtrMFS11	112327648	POPTR_005G015200v3	0	No	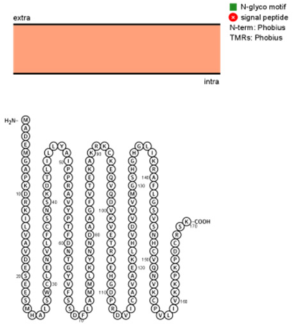

PtrMFS12	18098600	POPTR_005G018900v3	0	No	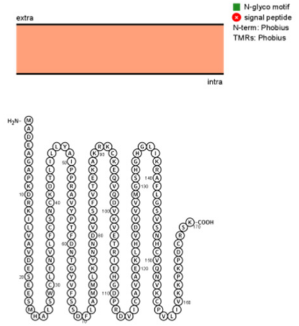
PtrMFS13	7469151	POPTR_005G177100v3	0	No	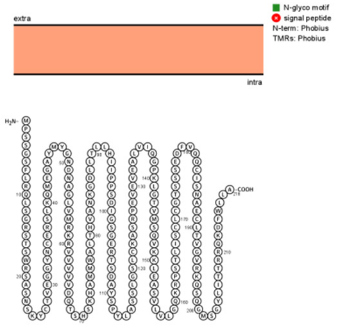
PtrMFS14	7454965	POPTR_006G092700v3	0	No	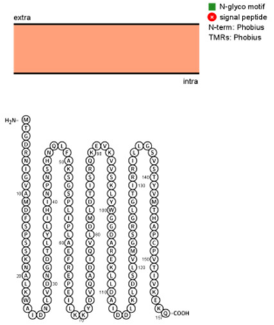

PtrMFS15	112327999	POPTR_006G225300v3	6	No	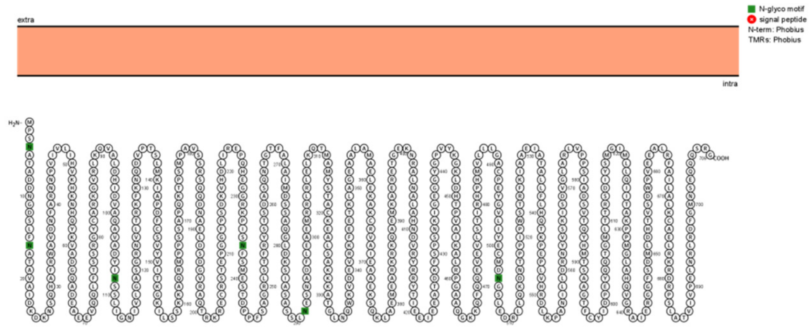
PtrMFS16	18100796	POPTR_006G279500v3	8	No	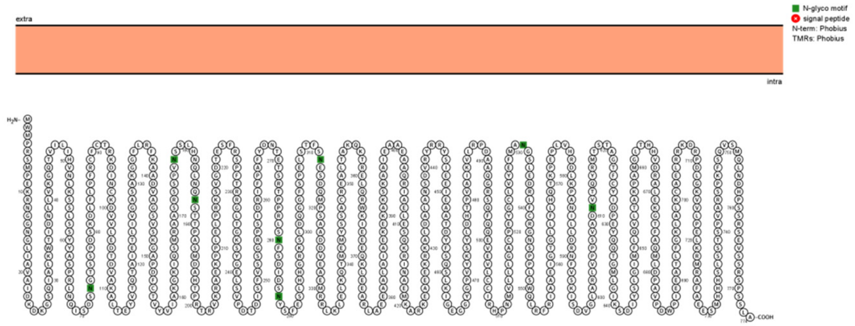
PtrMFS17	7483898	POPTR_008G109000v3	1	No	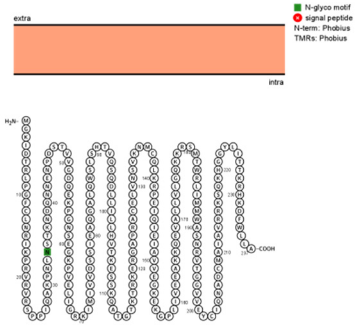

PtrMFS18	7488363	POPTR_008G121800v3	0	No	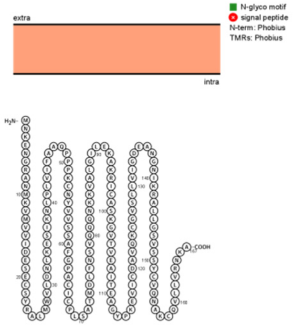
PtrMFS19	7488364	POPTR_008G121900v3	0	No	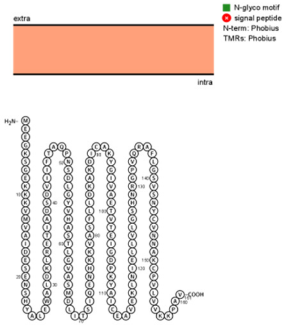
PtrMFS20	18101803	POPTR_008G221300v3	0	Yes	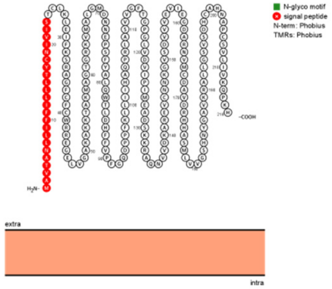

PtrMFS21	112328416	POPTR_008G226400v3	0	Yes	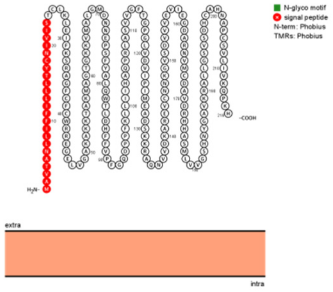
PtrMFS22	7464025	POPTR_009G117500v3	0	No	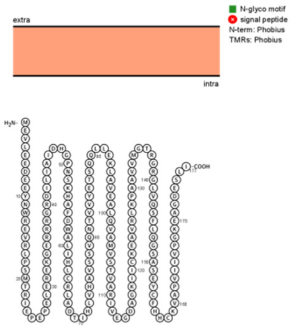
PtrMFS23	7475582	POPTR_010G123200v3	0	No	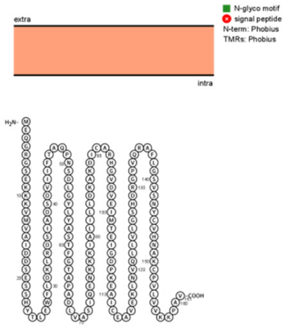

PtrMFS24	7475583	POPTR_010G123300v3	2	No	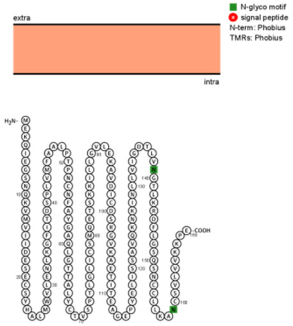
PtrMFS25	7475584	POPTR_010G123400v3	2	No	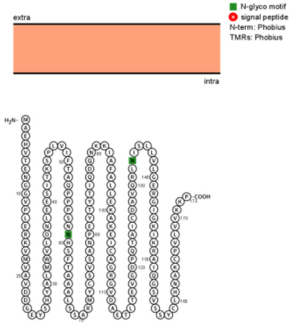
PtrMFS26	7482255	POPTR_010G140200v3	1	No	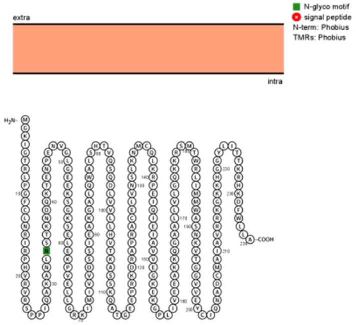

PtrMFS27	7468095	POPTR_010G144100v3	1	No	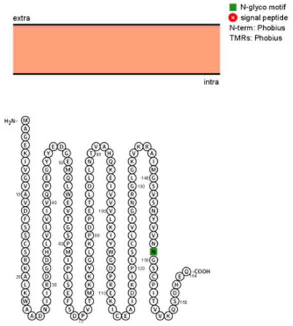
PtrMFS28	7495284	POPTR_011G039800v3	0	No	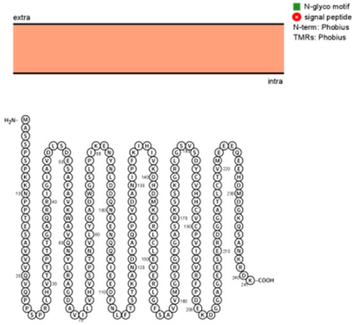
PtrMFS29	112323402	POPTR_011G125500v3	3	No	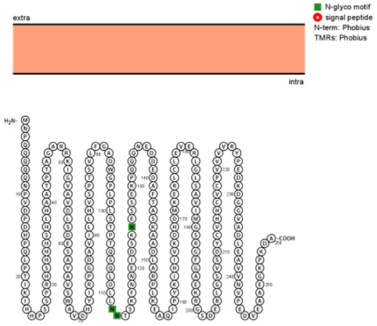

PtrMFS30	7487084	POPTR_012G059100v3	1	No	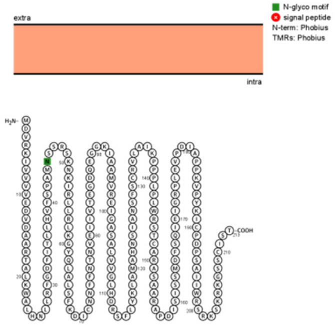
PtrMFS31	7458052	POPTR_012G084700v3	2	No	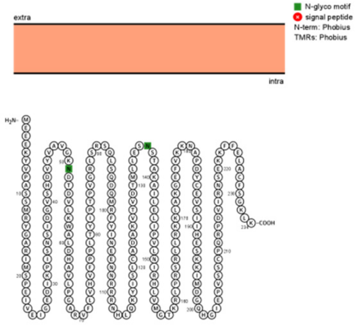
PtrMFS32	7481701	POPTR_013G009800v3	1	No	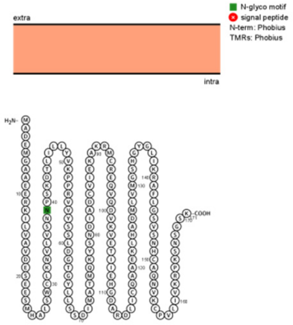

PtrMFS33	18104452	POPTR_013G112300v3	2	No	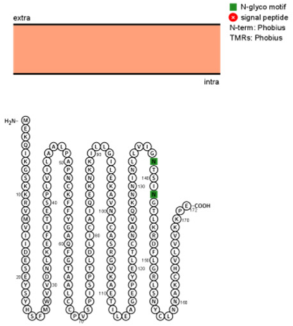
PtrMFS34	7494451	POPTR_013G150200v3	3	No	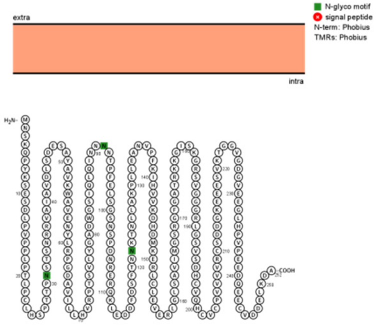
PtrMFS35	18109283	POPTR_014G122000v3	1	No	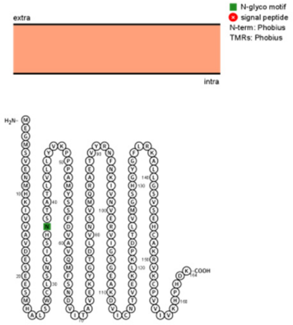

PtrMFS36	7491306	POPTR_014G130100v3	1	No	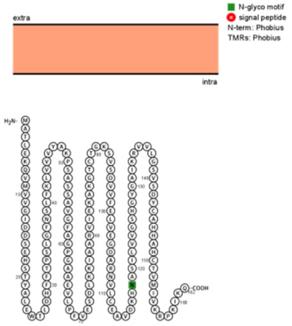
PtrMFS37	18105772	POPTR_015G060700v3	1	No	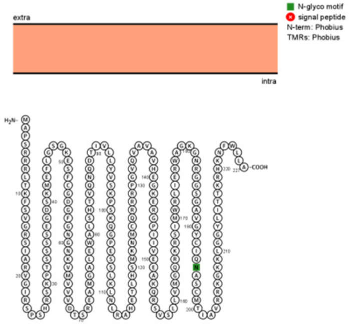
PtrMFS38	7453799	POPTR_015G083100v3	3	No	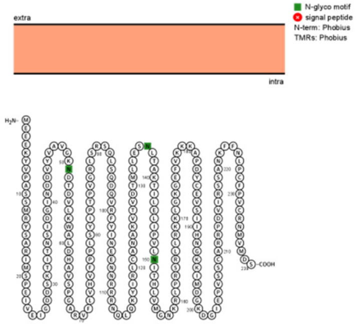

PtrMFS39	7488056	POPTR_016G064000v3	0	No	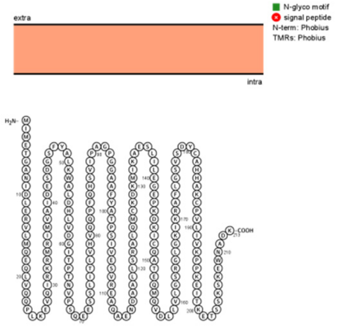
PtrMFS40	7486537	POPTR_016G104600v3	0	No	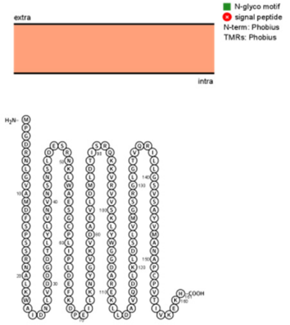
PtrMFS41	7496651	POPTR_017G071700v3	2	No	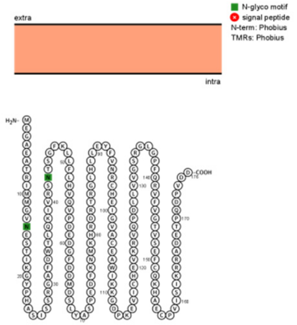

PtrMFS42	7463713	POPTR_018G061600v3	8	No	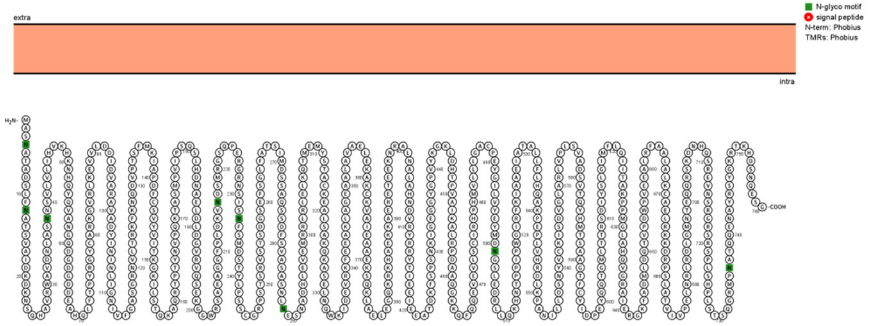
PtrMFS43	7458850	POPTR_019G119400v3	1	No	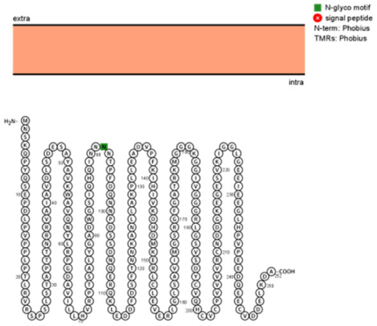
PtrMFS44	7460863	POPTR_T024200v3	3	No	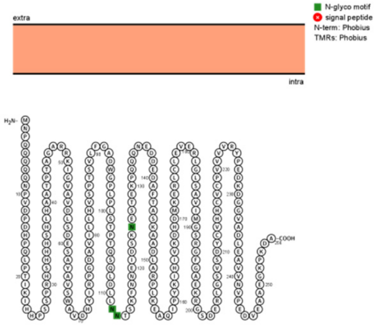

PtrMFS45	7496605	POPTR_T059500v3	3	No	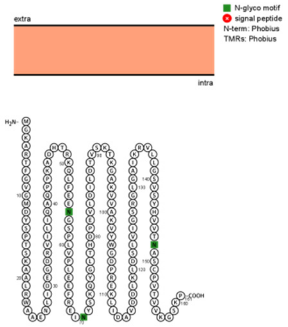
PtrMFS46	112325879	POPTR_T120500v3	1	No	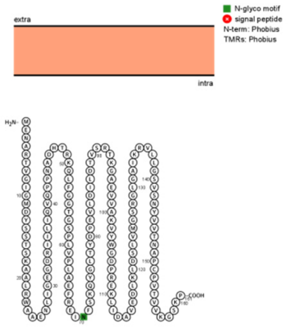
