# Supplementary material for: Comprehensive Analysis of Universal Stress Protein Family Genes and Their Expression in Fusarium oxysporum Response of Populus davidiana × P. alba var. pyramidalis Louche Based on the Transcriptome
Source: Int J Mol Sci. 2023 Mar 11;24(6):5405. doi: 10.3390/ijms24065405 (PMC10049587; doi:10.3390/ijms24065405)
Supplement: Supplementary file 1 [file ijms-24-05405-s001.zip › Table S6 Subcellular localization and gene coordinate of PtrUSPs.pdf]

Table S6. Subcellular localization and gene coordinate of PtrUSPs

Name in this paper	Gene ID	Locus tag	Subcellular localization	Chromosome
PtrUSP1	112326636	POPTR_001G409100v3	Nucleus	Chromosome 1
PtrUSP2	18095671	POPTR_001G414800v3	Nucleus	Chromosome 1
PtrUSP3	7466474	POPTR_002G084600v3	Nucleus	Chromosome 2
PtrUSP4	7461816	POPTR_002G104700v3	Cytoplasm	Chromosome 2
PtrUSP5	7481410	POPTR_002G193800v3	Cytoplasm	Chromosome 2
PtrUSP6	7481397	POPTR_002G196700v3	Cytoplasm	Chromosome 2
PtrUSP7	7487779	POPTR_002G205300v3	Cytoplasm	Chromosome 2
PtrUSP8	7494517	POPTR_004G075400v3	Cytoplasm	Chromosome 4
PtrUSP9	7469997	POPTR_004G156100v3	Cytoplasm	Chromosome 4
PtrUSP10	7461225	POPTR_004G156200v3	Cytoplasm	Chromosome 4
PtrUSP11	112327648	POPTR_005G015200v3	Cytoplasm	Chromosome 5
PtrUSP12	18098600	POPTR_005G018900v3	Cytoplasm	Chromosome 5
PtrUSP13	7469151	POPTR_005G177100v3	Chloroplast; Mitochondrion	Chromosome 5
PtrUSP14	7454965	POPTR_006G092700v3	Cytoplasm	Chromosome 6
PtrUSP15	112327999	POPTR_006G225300v3	Cytoplasm	Chromosome 6
PtrUSP16	18100796	POPTR_006G279500v3	Nucleus	Chromosome 6
PtrUSP17	7483898	POPTR_008G109000v3	Chloroplast; Mitochondrion	Chromosome 8
PtrUSP18	7488363	POPTR_008G121800v3	Peroxisome	Chromosome 8
PtrUSP19	7488364	POPTR_008G121900v3	Cytoplasm	Chromosome 8
PtrUSP20	18101803	POPTR_008G221300v3	Chloroplast	Chromosome 8
PtrUSP21	112328416	POPTR_008G226400v3	Chloroplast	Chromosome 8
PtrUSP22	7464025	POPTR_009G117500v3	Cytoplasm	Chromosome 9
PtrUSP23	7475582	POPTR_010G123200v3	Cytoplasm	Chromosome 10

PtrUSP24	7475583	POPTR_010G123300v3	Chloroplast	Chromosome 10
PtrUSP25	7475584	POPTR_010G123400v3	Chloroplast	Chromosome 10
PtrUSP26	7482255	POPTR_010G140200v3	Chloroplast_Mitochondrion	Chromosome 10
PtrUSP27	7468095	POPTR_010G144100v3	Cytoplasm	Chromosome 10
PtrUSP28	7495284	POPTR_011G039800v3	Chloroplast	Chromosome 11
PtrUSP29	112323402	POPTR_011G125500v3	Chloroplast; Cytoplasm	Chromosome 11
PtrUSP30	7487084	POPTR_012G059100v3	Cytoplasm	Chromosome 12
PtrUSP31	7458052	POPTR_012G084700v3	Cytoplasm	Chromosome 12
PtrUSP32	7481701	POPTR_013G009800v3	Cytoplasm	Chromosome 13
PtrUSP33	18104452	POPTR_013G112300v3	Nucleus	Chromosome 13
PtrUSP34	7494451	POPTR_013G150200v3	Cytoplasm	Chromosome 13
PtrUSP35	18109283	POPTR_014G122000v3	Cytoplasm	Chromosome 14
PtrUSP36	7491306	POPTR_014G130100v3	Cytoplasm	Chromosome 14
PtrUSP37	18105772	POPTR_015G060700v3	Mitochondrion	Chromosome 15
PtrUSP38	7453799	POPTR_015G083100v3	Nucleus	Chromosome 15
PtrUSP39	7488056	POPTR_016G064000v3	Cytoplasm	Chromosome 16
PtrUSP40	7486537	POPTR_016G104600v3	Nucleus; Cytoplasm	Chromosome 16
PtrUSP41	7496651	POPTR_017G071700v3	Chloroplast	Chromosome 17
PtrUSP42	7463713	POPTR_018G061600v3	Nucleus	Chromosome 18
PtrUSP43	7458850	POPTR_019G119400v3	Cytoplasm	Chromosome 19
PtrUSP44	7460863	POPTR_T024200v3	Chloroplast	-
PtrUSP45	7496605	POPTR_T059500v3	Cytoplasm	-
PtrUSP46	112325879	POPTR_T120500v3	Cytoplasm	-
