# Supplementary material for: Comprehensive Analysis of Universal Stress Protein Family Genes and Their Expression in Fusarium oxysporum Response of Populus davidiana × P. alba var. pyramidalis Louche Based on the Transcriptome
Source: Int J Mol Sci. 2023 Mar 11;24(6):5405. doi: 10.3390/ijms24065405 (PMC10049587; doi:10.3390/ijms24065405)
Supplement: Supplementary file 1 [file ijms-24-05405-s001.zip › Table S7 6 pairs repetitive events in PtrUSP genes and its KaKs ratio.pdf]

Table S7. 6 pairs repetitive events in PtrUSP genes and its Ka/Ks ratio.

Gene-1	Chromosome Location	Gene-2	Chromosome Location	Ka	Ks	Ka/Ks	Type
PtrUSP9	Chr04	PtrUSP22	Chr09	0.152070902	1.089435653	0.13958686	Segmental
PtrUSP12	Chr05	PtrUSP11	Chr05	0.012967689	0.028018822	0.46282061	Segmental
PtrUSP17	Chr08	PtrUSP26	Chr10	0.06401082	0.230047701	0.278250205	Segmental
PtrUSP23	Chr10	PtrUSP18	Chr08	0.51930131	1.713494158	0.303065702	Segmental
PtrUSP20	Chr08	PtrUSP21	Chr08	0.002001335	0.027589318	0.072540229	Segmental
PtrUSP9	Chr04	PtrUSP10	Chr04	0.159030179	1.330178269	0.119555539	Tandem
