# Supplementary material for: Comprehensive Analysis of Universal Stress Protein Family Genes and Their Expression in Fusarium oxysporum Response of Populus davidiana × P. alba var. pyramidalis Louche Based on the Transcriptome
Source: Int J Mol Sci. 2023 Mar 11;24(6):5405. doi: 10.3390/ijms24065405 (PMC10049587; doi:10.3390/ijms24065405)
Supplement: Supplementary file 1 [file ijms-24-05405-s001.zip › Table S8 Homologous relationships between PtrUSPs and other species.pdf]

**Table S8.** Homologous relationships between *PtrUSPs* and other species

| *Populus trichocarpa - Glycine max* | | *Populus trichocarpa - Eucalyptus grandis* | | *Populus trichocarpa - Solanum lycopersicum* | | *Populus trichocarpa - Arabidopsis thaliana* | |
|---|---|---|---|---|---|---|---|
| Potri.001G409100 | Glyma.06G255200 | Potri.002G084600 | Eucgr.F04356 | Potri.001G409100 | Solyc07g063680 | Potri.001G409100 | AT4G27320 |
| Potri.001G414800 | Glyma.06G255200 | Potri.002G104700 | Eucgr.F00412 | Potri.002G104700 | Solyc04g076200 | Potri.001G409100 | AT5G54430 |
| Potri.001G409100 | Glyma.12G146100 | Potri.002G205300 | Eucgr.K03471 | Potri.002G084600 | Solyc06g060360 | Potri.001G414800 | AT4G27320 |
| Potri.001G414800 | Glyma.12G146100 | Potri.004G075400 | Eucgr.F02899 | Potri.004G156100 | Solyc01g109710 | Potri.001G414800 | AT5G54430 |
| Potri.002G196700 | Glyma.02G013100 | Potri.004G156100 | Eucgr.G01684 | Potri.005G015200 | Solyc01g100370 | Potri.002G196700 | AT3G62550 |
| Potri.002G104700 | Glyma.02G213100 | Potri.005G177100 | Eucgr.F04356 | Potri.005G018900 | Solyc01g100370 | Potri.002G205300 | AT2G47710 |
| Potri.002G205300 | Glyma.02G155600 | Potri.006G092700 | Eucgr.B02732 | Potri.005G177100 | Solyc06g060360 | Potri.004G156100 | AT2G21620 |
| Potri.002G084600 | Glyma.06G112700 | Potri.006G225300 | Eucgr.C00670 | Potri.006G279500 | Solyc08g069180 | Potri.006G092700 | AT3G53990 |
| Potri.002G196700 | Glyma.10G013700 | Potri.006G279500 | Eucgr.C02137 | Potri.006G092700 | Solyc09g011660 | Potri.006G225300 | AT5G12000 |
| Potri.002G205300 | Glyma.10G018700 | Potri.008G121800 | Eucgr.B03009 | Potri.009G117500 | Solyc01g109710 | Potri.006G279500 | AT2G24370 |
| Potri.002G084600 | Glyma.13G080500 | Potri.008G121900 | Eucgr.B03008 | Potri.010G144100 | Solyc01g059930 | Potri.006G279500 | AT4G31230 |
| Potri.002G104700 | Glyma.14G181000 | Potri.009G117500 | Eucgr.G01684 | Potri.011G039800 | Solyc02g079410 | Potri.008G121900 | AT1G68300 |
| Potri.002G196700 | Glyma.19G215800 | Potri.010G123200 | Eucgr.B03008 | Potri.011G039800 | Solyc03g006680 | Potri.009G117500 | AT2G21620 |
| Potri.002G205300 | Glyma.19G211000 | Potri.010G123300 | Eucgr.B03009 | Potri.011G125500 | Solyc07g063680 | Potri.010G140200 | AT2G03720 |
| Potri.004G156100 | Glyma.04G012400 | Potri.010G123400 | Eucgr.B03009 | Potri.012G084700 | Solyc03g112330 | Potri.010G144100 | AT3G17020 |
| Potri.004G075400 | Glyma.14G211300 | Potri.010G123400 | Eucgr.G02310 | Potri.012G084700 | Solyc06g071725 | Potri.011G039800 | AT1G11360 |
| Potri.005G177100 | Glyma.04G250100 | Potri.010G140200 | Eucgr.G02426 | Potri.013G009800 | Solyc01g100370 | Potri.011G125500 | AT4G27320 |
| Potri.005G015200 | Glyma.10G147700 | Potri.011G039800 | Eucgr.E03674 | Potri.015G060700 | Solyc03g114820 | Potri.011G125500 | AT5G54430 |
| Potri.005G018900 | Glyma.10G147700 | Potri.012G059100 | Eucgr.B00688 | Potri.015G083100 | Solyc03g112330 | Potri.014G122000 | AT3G62550 |
| Potri.005G177100 | Glyma.14G148800 | Potri.012G084700 | Eucgr.K02804 | Potri.015G083100 | Solyc06g071725 | Potri.014G130100 | AT2G47710 |
| Potri.005G015200 | Glyma.20G098451 | Potri.013G150200 | Eucgr.E03674 | Potri.015G060700 | Solyc06g069420 | Potri.016G104600 | AT3G53990 |
| Potri.005G018900 | Glyma.20G098451 | Potri.014G130100 | Eucgr.H01151 | Potri.016G104600 | Solyc09g011660 | | |
| Potri.006G092700 | Glyma.01G106200 | Potri.014G130100 | Eucgr.K03471 | | | | |
| Potri.006G092700 | Glyma.03G073710 | Potri.015G060700 | Eucgr.B00635 | | | | |

| | | | |
|---|---|---|---|
| Potri.006G225300 | Glyma.04G053100 | Potri.015G083100 | Eucgr.K02804 |
| Potri.006G092700 | Glyma.07G140800 | Potri.016G104600 | Eucgr.B02732 |
| Potri.006G092700 | Glyma.18G190700 | Potri.016G064000 | Eucgr.K01888 |
| Potri.009G117500 | Glyma.04G012400 | Potri.019G119400 | Eucgr.E03674 |
| Potri.010G140200 | Glyma.02G121800 | | |
| Potri.010G144100 | Glyma.15G141000 | | |
| Potri.011G125500 | Glyma.12G146100 | | |
| Potri.012G059100 | Glyma.05G014100 | | |
| Potri.012G084700 | Glyma.05G041100 | | |
| Potri.012G084700 | Glyma.17G085200 | | |
| Potri.013G009800 | Glyma.10G147700 | | |
| Potri.013G009800 | Glyma.20G098451 | | |
| Potri.014G122000 | Glyma.02G013100 | | |
| Potri.014G130100 | Glyma.02G155600 | | |
| Potri.014G122000 | Glyma.10G013700 | | |
| Potri.014G130100 | Glyma.10G018700 | | |
| Potri.014G122000 | Glyma.19G215800 | | |
| Potri.014G130100 | Glyma.19G211000 | | |
| Potri.015G083100 | Glyma.05G041100 | | |
| Potri.015G083100 | Glyma.17G085200 | | |
| Potri.015G060700 | Glyma.17G081300 | | |
| Potri.016G104600 | Glyma.01G106200 | | |
| Potri.016G064000 | Glyma.02G167700 | | |
| Potri.016G104600 | Glyma.03G073710 | | |
| Potri.016G104600 | Glyma.07G140800 | | |
| Potri.016G104600 | Glyma.18G190700 | | |
